# A Dual-Role Amphiphilic Photosensitizer: Enhancing Structural Uniformity and Optical Properties of Langmuir Monolayers

**DOI:** 10.1021/acs.langmuir.5c06004

**Published:** 2026-05-13

**Authors:** Sarah Jasmin Finkelmeyer, Charlotte Mankel, Anna Elmanova, Konrad Hotzel, Zekai Ye, Andrea Dellith, Stefan Zechel, Kalina Peneva, Martin D. Hager, Ulrich S. Schubert, Martin Presselt

**Affiliations:** † 40096 Leibniz Institute of Photonic Technology (IPHT), Albert-Einstein-Str. 9, Jena 07745, Germany; ‡ Institute for Organic and Macromolecular Chemistry (IOMC), 9378 Friedrich Schiller University Jena, Humboldtstr. 10, Jena 07743, Germany; § Institute of Physical Chemistry, Friedrich Schiller University Jena, Helmholtzweg 4, Jena 07743, Germany; ∥ Sciclus GmbH & Co. KG, Moritz-von-Rohr-Str. 1a, Jena 07745, Germany; ⊥ Center for Energy and Environmental Chemistry Jena (CEEC Jena), Friedrich Schiller University Jena, Philosophenweg 7a, Jena 07743, Germany; # Jena Center for Soft Matter (JCSM), Friedrich-Schiller-University Jena, Philosophenweg 7, Jena 07743, Germany; ∇ Helmholtz Institute for Polymers in Energy Applications Jena (HIPOLE Jena), Lessingstr. 12-14, Jena 07743, Germany; ○ Helmholtz-Zentrum Berlin für Materialien und Energie, Berlin 14109, Germany

## Abstract

Amphiphilic π-electron systems form two-dimensional crystalline domains at fluid interfaces, resulting in gaps between the domains. Most such systems are based on linear π-electron backbones that absorb only in the UV irradiation, limiting their use in photoenergy conversion. We propose integrating the roles of plasticizer and photosensitizer into a single molecule to produce homogeneous, continuous, and light-harvesting membranes. Twisted perylenes can fulfill this dual function, and simple theoretical models can predict the densest packing of π-electron systems. We fabricated molecular monolayers comprising an amphiphilic π-conjugated oligo­(phenylene ethynylene) derivative (**OPE-NH**_**2**_) and a twisted perylene dye (**PMIDA-C**_**12**_) exhibiting broad visible-light absorption. Monolayer homogeneity was assessed by Brewster-angle microscopy and atomic force microscopy across a range of mixing ratios, and optical properties were probed by using photothermal deflection spectroscopy. Experimentally derived packing densities were compared with cross-sectional areas and aggregate structures predicted by quantum-chemical calculations. **OPE-NH**_**2**_ monolayers accommodated up to 14 mol % **PMIDA-C**_**12**_ while maintaining homogeneity and exhibiting a marked increase in visible-range absorption. At higher dye loadings, self-aggregation disrupted the layer uniformity. These results demonstrate that our twisted amphiphilic dye can act simultaneously as a plasticizer and a photosensitizer. In addition, we show that π-stacking in Langmuir monolayers can be quantified and predicted by combining image binarization with simple theoretical models.

## Graphical abstract

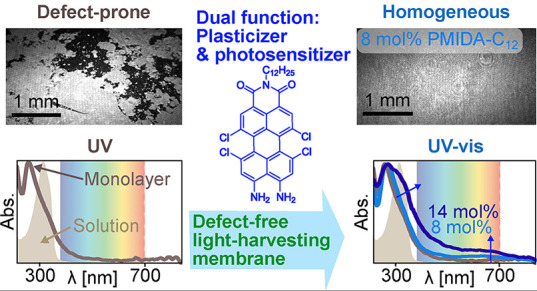

## 1 Introduction

Ultrathin organic semiconductor layers are of growing interest for separation^1^ and photocatalytic applications,^2–10^ including pollutant degradation^11–18^ and sustainable hydrogen generation.^2,4–7^ In particular, Langmuir monolayers composed of carefully designed and unidirectionally aligned molecular wires are ideally suited to control transmembrane charge transport, a key requirement for efficient photocatalytic water splitting.^19–21^ Through assembly of the amphiphilic and π-conjugated molecular building blocks at the air−water interface via the Langmuir technique densely packed, highly ordered, and intrinsically bifacial molecular monolayers can be obtained.^22–27^

Among the diverse π-conjugated materials^25–30^ established in molecular electronics, separation membranes, and photocatalysis,^31–34^ —including poly­(thiophenes),^35–37^ poly­(*p*-phenylenevinylenes),^38–40^ and poly­(fluorenes)^41–45^ −oligo­(phenylene ethynylene) (OPE)^46–50^ also called Oligo­(arylene ethynylene) (OAE) derivatives stand out as true workhorses for molecular electronics.^51^ Their extended π-electron system deliver exceptional high charge-carrier mobility, and efficient exciton transport,^52–60^ making them ideal for high-performance interfacial architectures.^46,49,50^ In particular, an OPE derivative bearing a terminal hydrophilic moiety^46^ (e.g., an amino group) and terminal hydrophobic moiety (e.g., a hydrophobic dodecyloxy chain), **OPE-NH**_**2**_,^26^ has demonstrated the ability to form uniformly aligned molecules at the air−water interface.

However, while strong π−π interactions are crucial for the electronic coupling of matrix or support material in processes such as photocatalytic hydrogen evolution,^61^ they simultaneously drive aggregation.^62,63^ In case of **OPE-NH**_**2**_, assembly at the air−water interface yields isolated crystalline two-dimensional (2D) domains separated by persistent gaps resulting from the rigidity of the domains.^26^ To mitigate this issue, molecular plasticizers have been recently introduced as effective additives that promote formation of uniform molecular layers by minimizing interfacial voids.^26^

For monolayers applied in light-driven applications and devices, such as water splitting or pollution degradation, it is essential that they incorporate photocatalytically active building blocks, e.g., photosensitizers, photocatalysts, or molecular dyads. Conventional plasticizers, as used in reference,^26^ offer no functional benefit beyond homogenizing the Langmuir monolayer. It would be advantageous to employ a component that not only acts as a plasticizer but also provides optical activity in the visible range, thereby improving and extending the film’s light absorption into this spectral region.

Dyes suited for this purpose must not only be miscible with the OPE matrix but also disrupt the crystalline packing sufficiently to reduce the domain rigidity. This requires amphiphilicity and a three-dimensional molecular structure. Bay-functionalized, twisted perylenes fulfill these criteria particularly well. Perylene-based chromophores are well-established in optoelectronic applications, particularly in organic photovoltaics,^64^ where they enhance charge transfer and electron mobility while improving long-term operational stability and morphological control of the active layer.^65,66^ Amphiphilic perylene additives have also been shown to optimize donor−acceptor network morphology, reduce unfavorable phase separation, and maintain high power conversion efficiency over extended operational periods.^65^

These findings suggest that incorporating an amphiphilic perylene-based additive into a Langmuir monolayer could simultaneously improve the film homogeneity and extend its optical absorption into the visible range. Previous study have revealed that only small amounts of plasticizers were needed to homogenize the monolayers while preserving the molecular alignment of OPEs.^26^ Based on these insights, the present work investigates the use of an amphiphilic perylene-based additive as a multifunctional plasticizer for **OPE-NH**_**2**_ monolayers, aiming to identify the optimal concentration for achieving homogeneous, continuous films and to assess its effect on visible-range optical absorption.

## 2 Experimental and Theoretical Details

### 2.1 Materials

The 4-((4-((4-(dodecyloxy)­phenyl)­ethynyl)­phenyl)­ethynyl)­aniline (**OPE-NH**_**2**_) and perylene monoimide diamine with C_12_-alkyl chain (**PMIDA-C**_**12**_) were synthesized according to the literature of Finkelmeyer et al.^26^ and Gerase et al.,^65^ respectively. The purity of each substance was confirmed by NMR (for **OPE-NH**_**2**_ see Finkelmeyer et al.^26^ and for **PMIDA-C**_**12**_ see SI section 5, page 34 and following).

### 2.2 Π­(mma) isotherm

For Π­(mma) isotherm characterization, we followed the procedure described by Finkelmeyer et al.,^26^ with minor modifications. Solutions of **OPE-NH**_**2**_, **PMIDA-C**_**12**_, and mixtures thereof (for molar mixing ratios see Table 1) were prepared in dichloromethane (Carl Roth, ROTISOLV, min. 99,8%, UV/IR-Grade) at a concentration of 0.1 μmol/mL and tempered to 35 °C 20−30 min prior spreading. Aliquots of 1500 μL were spread onto ultrapure-water subphase (0.1−0.25 μS/cm), water temperature controlled at 25 °C, in a Langmuir−Blodgett (LB) trough (KSV 5000, length = 520 mm, compression length = 475.2 mm, width = 150 mm). This corresponds to an average initial molecular area of 178 Å^2^ per molecule, calculated as the water-surface area divided by the number of spread molecules: ((475.2 × 150) × 10^14^ Å^2^)/(((0.1/1.5) × (10^6^)­mol) × 6.022 × 10^23^ (1/mol)). To allow complete evaporation of the solvent, 20 min are granted before starting barrier movement (maximum forward and backward rate of 10 mm/min, with further rate limitation of 5 mN/m/min—as long surface pressure is constant maximum rate applies, if the surface pressure rises the further rate limitation applies) and Π­(mma) isotherm recording. The defined and logged lateral velocity (millimeters per minute) allows the LB5000.bat software to calculate at each point of time the available water surface on which the molecules are assembled. Three to four Π­(mma) isotherms were recorded and averaged, and error bands were calculated. The surface compression modulus *C* ^−^^1^_s_ was calculated from the averaged Π­(mma) isotherm by applying the formula $C_{S}^{-1}=-A\times \left(\frac{d\Pi }{\mathrm{dmma}}\right)$ .^67^ A_0_ was determined by extrapolating the steepest slope of the Π­(mma) isotherm (in the range of *C* ^−^^1^_s_,_max_).

**Table 1:** Molar Mixing Ratios **OPE-NH**_**2**_−**PMIDA-C**_**12**_

**molar mixing ratio [%]**	
**OPE-NH**_**2**_	**PMIDA-C**_**12**_
100	0
99	1
98	2
97	3
96	4
95	5
94	6
92	8
90	10
86	14
0	100

### 2.3 Brewster Angle Microscopy (BAM)

For Brewster angle microscopy (BAM)^26^ recordings of **OPE-NH**_**2**_, **PMIDA-C**_**12**_, and mixtures thereof, the solution parameters were kept the same (*c*_solution_ = 0.1 μmol/mL, *t*_solution_ = 35 °C) as well as the compression parameters, but the spread volume was increased to 2500 μL. The BAM images were captured with a KSV NIMA MicroBAM (Biolin Scientific). In the gas phase of the Π­(mma) isotherm, BAM images were taken every 2.5 mN/m. Thereafter, BAM images were recorded at 2, 4, 6, 8, 10, 15, and 20 mN/m as well as after holding at 20 mN/m for 5, 10, 15, and 20 min. For the pristine compounds and the mixtures thereof, the Langmuir monolayer formation was monitored once by BAM. At 20 mN/m the Brewster angle microscope was moved around, and images of the Langmuir film were taken at 12−16 different positions.

### 2.4 Langmuir Layer Deposition

For deposition of the Langmuir monolayers of **OPE-NH**_**2**_, **PMIDA-C**_**12**_, and mixtures thereof, the solution parameters were kept the same (*c*_solution_ = 0.1 μmol/mL, *t*_solution_ = 35 °C) as well as the compression parameters, but the spread volume was between 1200 and 1800 μL. The monolayers were compressed to 20 mN/m, and a surface pressure of 20 mN/m was kept (hold) for 20 min. Two different types of deposition technique were used—the horizontal Langmuir−Schaefer (LS) for the monolayer preparation and the rolling transfer Langmuir layer (rtLL)^25^ deposition technique for the multilayer preparation. The LS deposition of the Langmuir monolayers was performed onto single side polished silicon substrates and quartz glass substrates hydrophobized with stearic acid (Fluka Analytical, analytical standard, ≥99.5% purity, Sigma-Aldrich) self-assembled monolayers from a 5 mM ethanol (Rotisolv HPLC gradiant grade, ≥99.9% purity, Carl Roth) solution for AFM and photothermal deflection spectroscopy (PDS), respectively. On quartz glass substrates hydrophobized with stearic acid, multilayer stacks of 4 layers were prepared by using the rtLL deposition technique.

### 2.5 Atomic Force Microscopy (AFM)

AFM analysis was performed on a Dimension Edge from Bruker according to the procedures described in Gerase et al.^65^ and in Finkelmeyer et al.^25^ The silicon tip has a radius of approximately 10 nm and was used in tapping mode with a scan frequency (with respect to the speed in reading lines) of 0.4 Hz. Resolution was 512 pixels × 512 pixels. Post data processing was performed using Gwyddion (version: 2.61) software, and the following steps were applied sequentially: “base flatten”, “align rows” with the selection mean of differences, “remove scars”, “polynomial background”, and set minimum value as zero point (“fix zero”). Height profiles (horizontal lines) of 1 pixel thickness were generated. The color scale was used in a fixed range. The Langmuir monolayers were LS-type deposited onto single side polished silicon substrates. AFM analysis was performed on two different spots for each sample Each spot was scanned using scan windows of 100 μm × 100 and 20 μm × 20 μm, respectively.

### 2.6 Photothermal Deflection Spectroscopy (PDS)

The photothermal deflection spectroscopy (PDS)^68–73^ was performed on the same setup as used in Smirnova et al.^74^ The whole system is controlled by a self-written Labview program,^75,76^ which collects all data and corrects the PDS signal according to the incident light intensity. A glassy carbon plate (3 mm × 30 mm) was used as the reference sample. The samples, −1 LS monolayers or 4 rtLLs on hydrophobized quartz glass, were fixed in the center of a quartz glass cuvette (CV10Q3500F, Thorlabs) filled with FC-40 (Sigma-Aldrich). The PDS measurement was carried out from 200 to 1200 nm in 4 nm steps with the monochromator slits at 3.2 mm. The resolution was 8 nm in the wavelength range 200 to 1000 and 16 nm in the wavelength range 1000−1600 nm.

### 2.7 Quantum Chemical Calculation

Quantum chemical structure optimizations^77–79^ along with the calculations of electrostatic potentials and the cross sectional area determination were carried out accordingly to quantum chemical calculations as in Finkelmeyer et al.^26^

### 2.8 Image Binarization and mma_domain_ Determination

The replotted data from Finkelmeyer et al.^26^ in Figure 2a was calculated by applying thresholding binarization to the BAM images and subsequent deriving the reduced mmas of the amphiphiles within the Langmuir domains (mma_domain_) as detailed in Finkelmeyer et al.^26^

## 3 Results and Discussion

### 3.1 Homogeneity of Langmuir Monolayers

In this study, we investigate the amphiphilic perylene monoimide-diamine (**PMIDA-C**_**12**_), bearing two amine groups at the peri positions, four chloro substituents at the bay positions, and a dodecyl chain on the imide, which imparts amphiphilic character (cf. Figure 1a). This substitution pattern produces a push−pull chromophore, and the electrostatic potential mapping of the solvent-accessible surface illustrates its molecular polarity distribution. The second component, **OPE-NH**_**2**_, forms rigid Langmuir monolayer domains at the air−water interface (cf. Figure 1c,i). It consists of a linearly extended π-electron system, an anchoring amine group, and a hydrophobic dodecyloxy (−OC_12_H_25_) substituent. As shown in the Brewster angle microscopy (BAM) images in the bottom row of Figure 1, compression to 20 mN/m yields extended, micrometer-sized crystalline domains that remain separated by persistent interstitial gaps. These gaps do not close upon further lateral compression, indicating limited domain flowability.

Based on these characteristics, we hypothesized that incorporating **PMIDA-C**_**12**_ into **OPE-NH**_**2**_ Langmuir monolayers would serve a dual role: (i) provide visible-range optical absorption and (ii) plasticize the rigid **OPE-NH**_**2**_ domains, increasing their flowability so they could reorganize and fill the gaps between individual domains. This structural reorganization would, in turn, promote the formation of a continuous, homogeneous Langmuir monolayer. When small amounts of **PMIDA-C**_**12**_ (structure shown in Figure 1a), which we introduce here as dual-function plasticizer, are incorporated into **OPE-NH**_**2**_ monolayers, a clear improvement in film continuity is observed as shown in Figure 1f−h. Upon lateral compression, the previously isolated **OPE-NH**_**2**_ domains begin to coalesce, forming a denser, more homogeneous monolayer with significantly reduced voids. The BAM image in Figure 1f, as well as the results for additional mixing ratios (see Supporting Information), consistently show that a content of 8 mol % **PMIDA-C**_**12**_ in **OPE-NH**_**2**_ leads to the most uniform and seemingly defect-free Langmuir monolayer at 20 mN/m, thus almost reaching the layer homogeneity achieved with a the bisphenol A di*tert*-butyl ester (BPAE)-plasticizer employed earlier, which does not show any absorption in the visible spectral range.^26^

**Figure 1:**
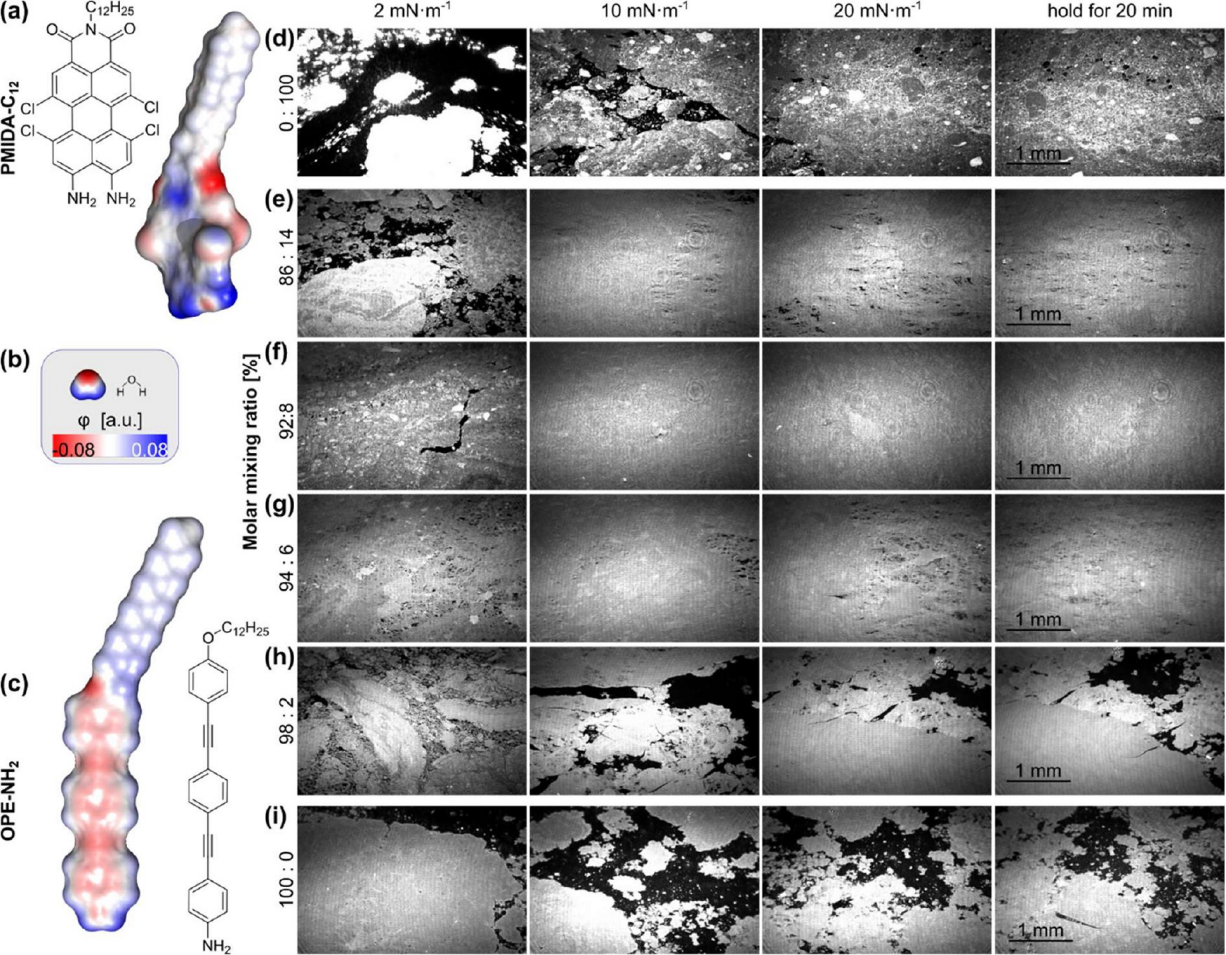
Panel (a−c) Lewis structures and electrostatic potential (φ) distributions derived via density functional theory **OPE-NH**_**2**_ and **PMIDA-C**_**12**_; Water is used as reference to determine the color scale limits (φ= ±0.08 au) for all φ-representations to highlight polar parts. Panel (d−i) Overview of Brewster Angle Microscopy (BAM) images of pristine **OPE-NH**_**2**_ in (i) and of pristine **PMIDA-C**_**12**_ in (d). (e−h) of mixtures of **OPE-NH**_**2**_ and **PMIDA-C**_**12**_.

### 3.2 Rigidity and Packing Density of Langmuir Monolayers

The surface pressure (Π) vs. mean molecular area (mma) isotherms of the pure compounds **OPE-NH**_**2**_ and **PMIDA-C**_**12**_, as well as their mixtures containing 1 to 14 mol % **PMIDA-C**_**12**_, are shown in Figure 2a. The Π­(mma) isotherm of **PMIDA-C**_**12**_ is shifted to larger mmas than the Π­(mma) isotherm of **OPE-NH**_**2**_, according to the larger spatial demand of **PMIDA-C**_**12**_ as compared to **OPE-NH**_**2**_. The steep slope of the Π­(mma) isotherms indicates that the Langmuir monolayers, in both pure and mixed films, are highly rigid. This is supported by the exceptionally high maximum compression moduli (Figure 2b), calculated using *C*_S_^−1^= −A dΠ/dA, which exceed 150 mN/m.^80–89^ For **PMIDA-C**_**1**_ **_2_**, the maximum compression modulus is reached at a mean molecular area (mma_C_) of 39 Å^2^, while for the **OPE-NH**_**2**_ monolayer and all blends mma_C_ is determined at 27 Å^2^.

Considering the holes observed in the BAM images of the pure **OPE-NH**_**2**_ monolayer, the local two-dimensional packing density within the dense domains must be higher than the average density across the entire film. Consequently, the mean molecular area within the domains (mma_domain_) is smaller than the global mma_C_. Using a recently introduced binarization method applied to the BAM images,^26^ we determined the mean molecular area within the domains mma_domain_(**OPE−NH**_**2**_) to be 23 Å^2^, as shown in Figure 2a. This value closely matches the theoretically calculated cross-sectional area of the **OPE-NH**_**2**_ amphiphile if it is vertically aligned at the air−water interface (see Figure 2c−h), thus supporting the conclusion of dense molecular packing at 1/mma_domain_ = 4.3 molecules/nm^2^.

The comparison between the experimental and theoretical cross-sectional areas is based on two theoretical methods that determine the minimum and maximum space requirements for the molecules. As shown in Figure 2, the maximum space requirement is determined by using bounding boxes around the molecules. Naturally, there is a lot of empty space in the resulting box, meaning that the smallest area is significantly larger than the experimentally determined mma_C_ value.

The cross-sectional areas determined by the molecular surface (more specifically, the solvent-excluded surface or SES) were calculated along the *z*-axes of the vertically oriented amphiphiles, resulting in the *xy* cross-sectional area profiles shown in Figure 2e,f. For the **OPE-NH_2_** amphiphile, the SES xy cross-sectional areas was determined to be approximately 18−19 Å^2^. These cross-sectional areas can be approximated as elliptical. With a moderate eccentricity of the ellipses, their densest packings do not fill the space. In the limiting cases of circles (*A*_circle_ = π*r* ^2^) and their densest (hexagonal) packing (*A*_unitcell_ = (2r)^2^sin60°), the ratio *A*_unitcell_/*A*_circle_ = (4*r* ^2^sin60°)/(π*r* ^2^) = 4 sin60°/π ≈ 1.10. The following applies to circles and ellipses: *A*_unitcell_ = 4/π sin­(θ) *A*_circle/ellipse_.

The prefactors range from 1.10 (θ = 60°, hexagonal, closest packing) to 1.27 (θ = 90°, rectangular packing). Multiplying the largest minimum cross-sectional area values (Figure 2e,f) by 1.2 for an average packing density gives a theoretical area requirement of approximately 23 Å^2^, which is consistent with the value determined for the mma_domain_ by combining BAM image binarization and isothermal evaluation.^26^

**Figure 2:**
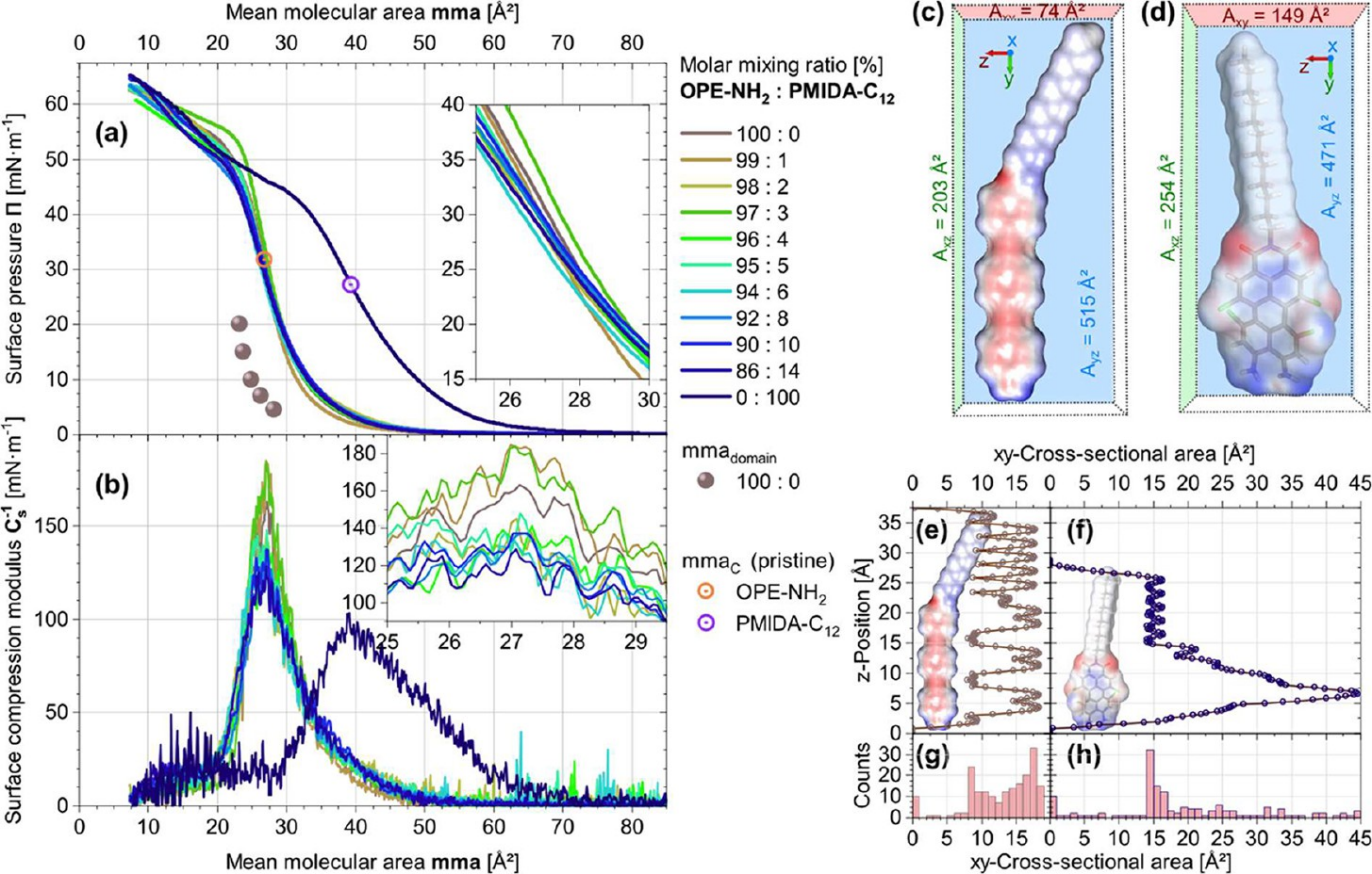
**OPE-NH**_**2**_, dual function molecule (**PMIDA-C**_**12**_) (plasticizer and PS), and its mixtures. (a) Π­(mma) isotherms and (b) surface compressional moduli of *C*_s_^−1^. (c) and (d) **OPE-NH**_**2**_ and **PMIDA-C**_**12**_, respectively, in binding box and areas of the three binding box planes. (e) and (f) *xy*-cross sectional plane scan along *z*-axis for **OPE-NH**_**2**_ and **PMIDA-C**_**12**_ solvent excluded surface, respectively. Panels (g) and (h) present the distributions of the *xy*-cross-sectional area along the *z*-axis at the van der Waals surface for the **OPE-NH**_**2**_ and **PMIDA-C**_**12**_ molecules, respectively. The plotted spheres in panel a, the mma_domain_ data, are from Finkelmeyer et al.^26^

These two simple methods (via SES together with lateral packing densities and via bounding boxes) yield upper and lower bounds for spatial demands. For geometrically simple amphiphiles such as those studied here, the surface area determined using SES corresponds perfectly with the experimentally determined reciprocal packing densities obtained from BAM image binarization.^26^ For more complex molecular structures, either the local resolution of the cross-sectional profiles can be exploited or packing densities in aggregates can be calculated using more complex methods such as predicting explicit aggregate structures and calculating their spatial demand.

Following the latter approach, we generated dimers and larger aggregates by systematically adding further molecules. This is followed by geometry optimization, similarity analysis of the optimized structures, reduction of the set of aggregates generated in this way by forming geometric families, and consideration of only the most energetically favorable representatives of these families, as shown in section 4 of the SI. The quadrumers having a relative Boltzmann probability of greater than 10% are shown in Figure 3b. To estimate the lateral space requirement, we defined a “footprint” area as the polygon spanned by the four per-molecule centers of mass after projection to their best-fit plane, as illustrated in Figure 3e with the polygon overlaid. The obtained areas for not sandwich-stacked quadrumers range from 13.36 to 30.02 Å^2^, as seen in Figure 3c and thus also fit perfectly with the experimentally determined exact mma_domain_ areas.^26^

**Figure 3:**
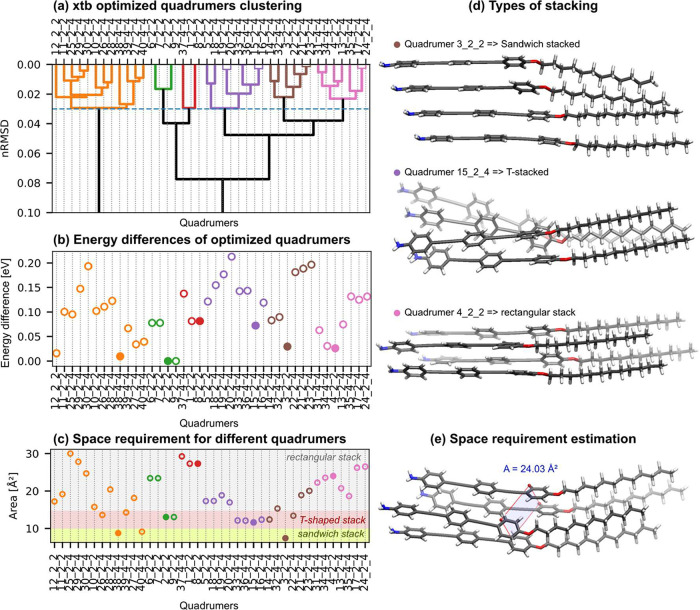
Quadrumer motifs, energies, and unit cell areas. Panel (a): Hierarchical clustering of all of the considered quadrumers. The dashed blue line indicates a nRMSD cutoff of 0.03 Å. Panel (b): Energies differences ΔE (meV) between quadrumers (sorting and coloring identical to the dendrogram above). Energies are referenced to the global minimum. Panel (c): Unit-cell areas (Å^2^) as determined by the centers of mass of the monomers composing the quadrumers. Shaded bands highlight different packing motifs: sandwich (small area), T-shaped (intermediate area), and rectangular (large area). Panel (d): Representative low-energy geometries from distinct families: a sandwich stack, a T-stacked, and a rectangular stack. Panel (e): Example area measurement for a rectangular stack (here 24.03 Å^2^). Quadrumer labels encode the dimer-motif sequence used in assembly (e.g., 4 means quadrumer 4 and 2_2 means It was made from 2nd dimer duplicated twice).

As reported for the BPAE plasticizer,^26^ one might expect the Π­(mma) isotherms to shift toward the lower mma_domain_ values if adding a plasticizer closed the gaps in the **OPE-NH**_**2**_ Langmuir monolayer. However, as Figure 2a shows, the Π­(mma) isotherms of the mixtures display no such shift compared to the pure **OPE-NH**_**2**_ Π­(mma) isotherm. We attribute the virtually constant Π­(mma) isotherm positions to the comparatively large cross-sectional area of **PMIDA-C**_**12**_ (mma_C_ = 39 Å^2^; see Figure 2b), that exceeds the one of BPAE.^26^ Thus, although **PMIDA-C**_**12**_ promotes greater film homogeneity by facilitating gap closure, its large spatial demand likely compensates for any reduction in the mean molecular area of **OPE-NH**_**2**_, resulting in an overall unchanged Π­(mma) isotherm position.

As shown in Figure 2a, the Π­(mma) isotherms of the mixed monolayers exhibit subtle variations in the slope. For 1 and 3 mol % of **PMIDA-C**_**12**_, the slope increases, indicating an enhanced compressional modulus. However, as the **PMIDA-C**_**12**_ content increases from 4 to 14 mol %, the slope of the isotherm decreases slightly. This reflects the softening of the Langmuir monolayers induced by the plasticizer and the resulting increase in the film homogeneity. This softening is further evidenced by a reduction in the maximum compressional modulus of about 30 mN/m at plasticizer contents above 4 mol % (cf. inset of Figure 2b).

At 25 mN/m, the mixture containing 6 mol % **PMIDA-C**_**12**_ has the smallest mean molecular area of the investigated Langmuir monolayers, as shown in Figure 2a. Nevertheless, BAM imaging revealed holes in this film (Figure 1). In contrast, the mixture containing 8 mol % shows very few visible holes, indicating nearly complete surface coverage and pronounced improvement in softening the **OPE-NH**_**2**_ monolayer. This promotes domain coalescence and hole filling.

### 3.3 Topography of Deposited Langmuir Monolayers

We deposited dense Langmuir monolayers close to mma_C_ at 20 mN/m after 20 min hold onto silicon substrates via the Langmuir−Schaefer technique to characterize the monolayer morphology via atomic force microscopy (AFM). The topography image of the pure **OPE-NH**_**2**_ Langmuir−Schaefer (LS) film shown in Figure 4 reveals a few sharp edges due to slipped, rigid, two-dimensional **OPE-NH**_**2**_ domains. This corresponds perfectly to the crystalline two-dimensional domains of **OPE-NH**_**2**_ discussed above. Additionally, there are minute particulate structures that we attribute to **OPE-NH**_**2**_ aggregates.

The edge of the pure **OPE-NH**_**2**_-LS film shown in the top left corner of Figure 4 is ideal for determining the film thickness. The determined step height of 3.5 nm matches the expected height of 3.6 nm, which is the length of the **OPE-NH**_**2**_ molecules (Figure 2). Detailed scans also reveal nanoscopic holes, whose depth corresponds to the film thickness.

The LS film of the pure plasticizer has a carpet-like texture with elongated voids, as shown at the bottom of Figure 4. The thickness of this film of the pure plasticizer can be determined at these voids to be 2.6 nm. This relatively homogeneous carpet-like structure is partially covered by flat layers. As the plasticizer content increases in the **OPE-NH**_**2**_-LS-monolayers, weak textures develop, indicating the formation of a second phase and wrinkling. Overall, the LS films that are quite rigid, as they have been deposited at their maximum compression moduli become more homogeneous with the addition of plasticizers and show fewer defects, confirming the success of our approach.

Finally, the 94:6 mixture (6 mol % **PMIDA-C**_**12**_) already exhibits a smooth and homogeneous topography, while the 92:8 mixture (8 mol % **PMIDA-C**_**12**_) yields the first truly defect-free monolayer. However, AFM recordings reveal early signs of upfolding, indicating a delicate balance between improved lateral homogeneity and the onset of vertical structural perturbations. Further increases in **PMIDA-C**_**12**_ content result in pronounced vertical molecular displacement and a loss of overall film integrity.

**Figure 4:**
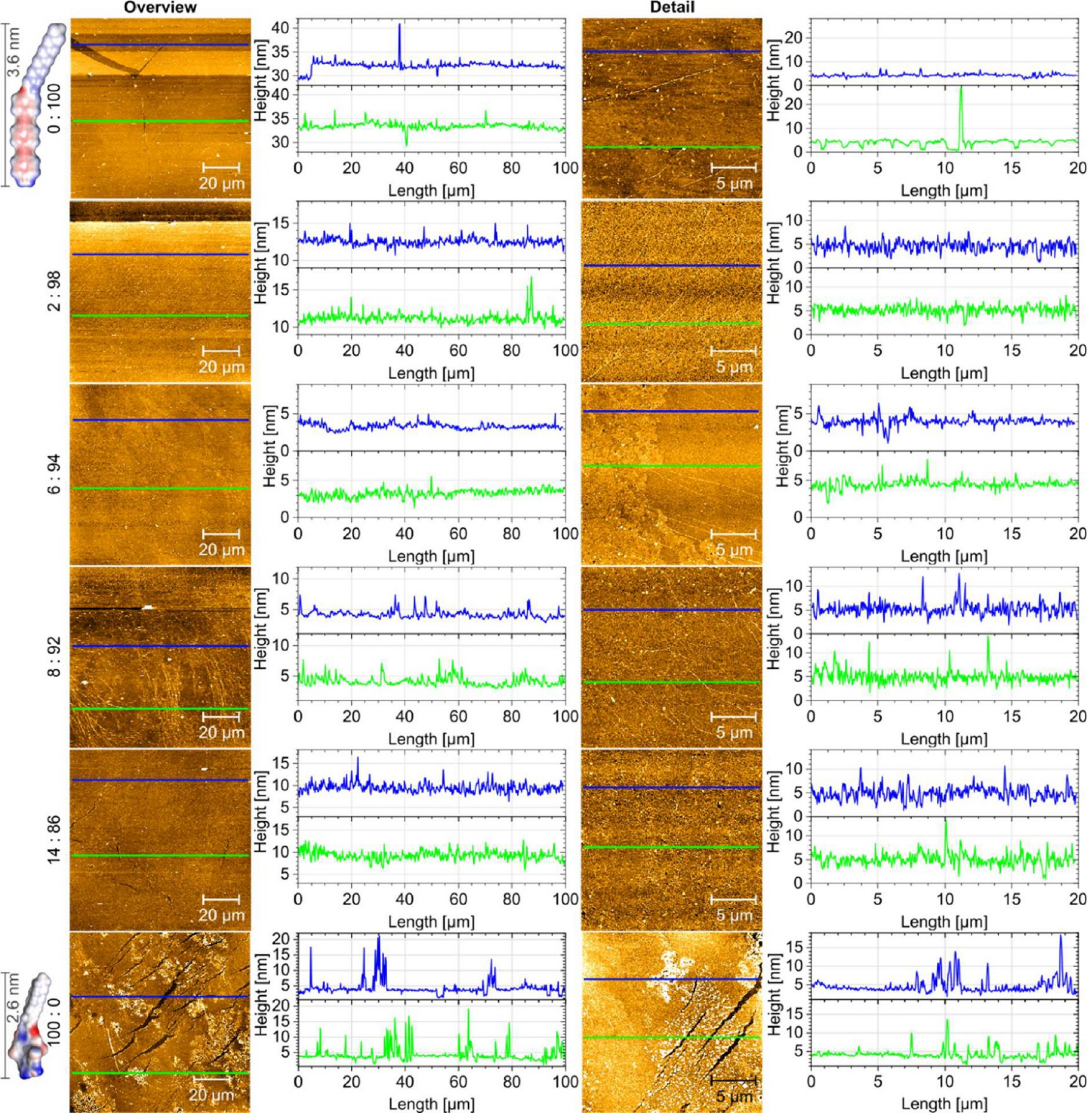
Atomic force microscopy (AFM) images of Langmuir−Schaefer deposited monolayers of **OPE-NH**_**2**_, **PMIDA-C**_**12**_ and mixtures thereof onto silicon substrates. Surface pressure at deposition was 20 mN/m.

### 3.4 UV−Vis Absorption Spectra of Deposited Layers

To evaluate the spectroscopic properties of the monolayers, we performed UV−vis transmission and photothermal deflection (PDS)^59,75,76^ spectroscopy to detect even the weak absorption^56,90,91^ in the molecular films. For reference, we also recorded the absorption spectra of **OPE-NH**_**2**_ dissolved in methylene chloride. These solution spectra closely resemble those of an OPE-benzoic acid derivative in hexane reported in literature.^92^ As illustrated in Figure 5, the **OPE-NH**_**2**_ solution exhibits an absorption maximum at 340 nm and a shoulder at 360 nm (see Figure S1 for spectra at different dilutions), which we attribute to vibrational progression. In contrast, the PDS spectrum of the pure **OPE-NH**_**2**_ LS-monolayer deposited onto a quartz glass is clearly blue-shifted, showing a maximum at 288 nm and a shoulder at 360 nm. According to literature, this hypsochromic shift indicates two-dimensional H-type aggregation of the **OPE-NH**_**2**_ chromophores within the monolayer.^27,86,87^ Consequently, the chromophores and transition dipole moments in the monolayer are aligned coparallel to each other.^46,92–94^

**Figure 5:**
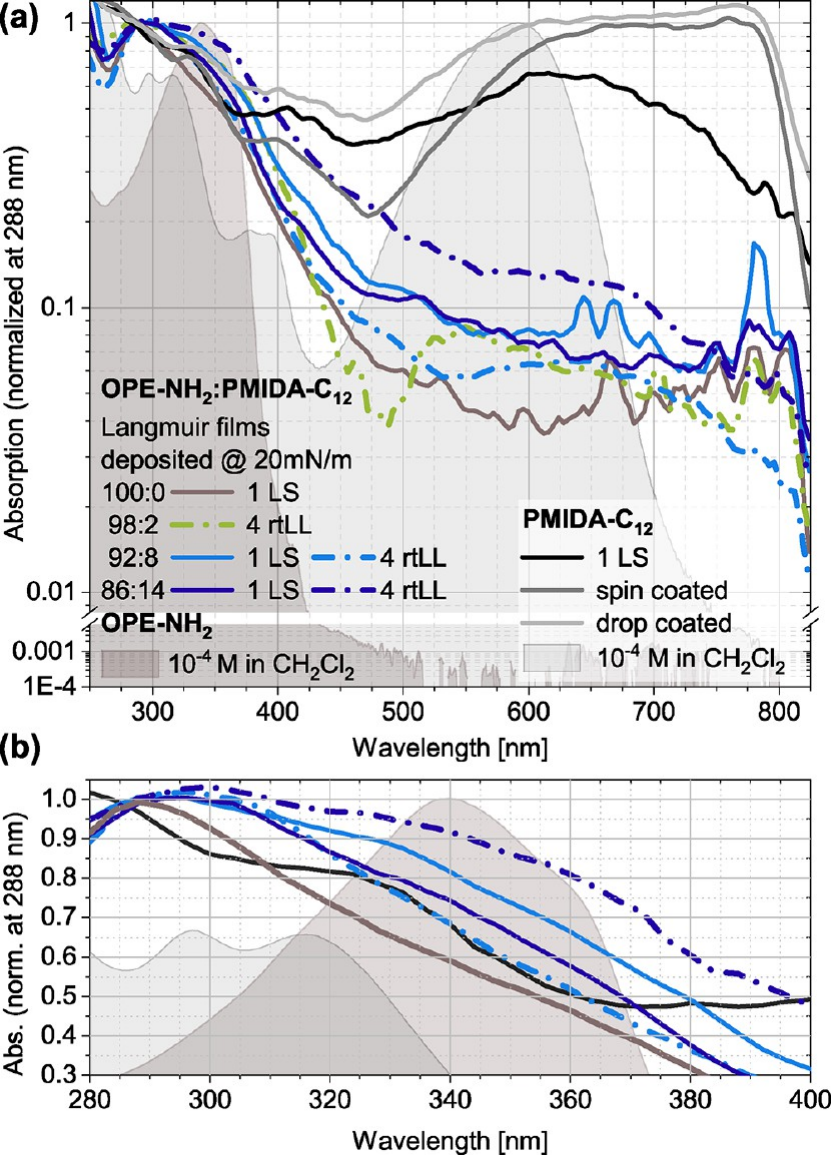
Absorption via Photothermal Deflection Spectroscopy (PDS) of LS (Langmuir−Schaefer) and rtLL (rolling transfer Langmuir layer) deposited monolayers on stearic acid self-assembled monolayer modified quartz glass substrates. Deposited Langmuir and other thin films normalized at 288 nm; solution references normalized at maximum. (a) overview. (b) selection (280−400 nm) enlarged.

As shown in Figure 5a, **PMIDA-C**_**12**_ dissolved in dichloromethane displays a broad absorption band spanning most of the visible range with a maximum at 590 nm. In the LS film, this band broadens further toward longer wavelengths, exhibiting a red-shifted, broad maximum compared to the solution spectrum. The observed spectral broadening suggests the presence of various aggregate species. The overall red shift indicates that the aggregates are predominantly J-type, arising from various head-to-tail molecular arrangements.^27,87,95^ Notably, spin-coated and drop-cast films exhibit an additional absorption peak at approx. 760−770 nm, indicating the formation of a dominant J-aggregate species absent from the more ordered LS monolayer.^96^

For mixed LS-monolayers, the **PMIDA-C**_**12**_ absorption signal is too weak to be distinguished from noise at ≤14 mol % loading. However, as shown in Figure 5, multilayer stacks (four Langmuir layers transferred with the rolling technique)^25^ with 8 and 14 mol % **PMIDA-C**_**12**_ show a distinct absorption in the red spectral range, which resembles the broad absorption peak of the pristine **PMIDA-C**_**12**_ LS monolayer. Consequently, regarding the visible spectral range, we achieve higher−additive−absorption of the **OPE-NH**_**2**_ layer by adding the **PMIDA-C**_**12**_ plasticizer.

Furthermore, adding **PMIDA-C**_**12**_ broadens the main absorption band of the **OPE-NH**_**2**_ LS layer toward longer wavelengths. This results in an absorption shoulder that coincides with the major absorption peak of the **OPE-NH**_**2**_ solution. Therefore, the expansion of the **OPE-NH**_**2**_ LS monolayer’s absorption peak is attributed to **PMIDA-C**_**12**_’s plasticizing role, which partially disrupts the tight aggregation of the **OPE-NH**_**2**_ amphiphiles within the monolayer. Consequently, the spectrum reflects a superposition of absorption from both aggregated and nonaggregated **OPE-NH**_**2**_ chromophores. These observations confirm **PMIDA-C**_**12**_’s dual function: It softens the rigid, defect-prone **OPE-NH**_**2**_ monolayer, promoting the formation of more homogeneous films, and enhances absorption in the visible spectral range.

## 4 Conclusion

In this work, we demonstrate a new concept for designing dual-function amphiphilic additives that act simultaneously as plasticizers and photosensitizers within Langmuir monolayers. By incorporating a twisted perylene dye (**PMIDA-C**_**12**_) into π-conjugated **OPE-NH**_**2**_ monolayers, we achieved unprecedented homogeneity and extended visible-light absorption in molecular membranes. Compared with our previous introduction of a nonabsorbing plasticizer,^26^ the present approach increases the permissible loading (to 8−14 mol %) while introducing substantial visible-range absorption, thereby integrating light harvesting and structural plasticization in a single molecule—an innovation that addresses a long-standing limitation in colloid and interface science.

We show that **PMIDA-C**_**12**_ closes interstitial gaps in rigid **OPE-NH**_**2**_ domains without significantly compromising monolayer order up to an optimal loading of 8 mol %. Beyond this threshold, self-aggregation perturbs the film, revealing a clear structure−property limit. This performance surpasses that of the BPAE plasticizer reported previously^26^ by combining (i) improved mixing and domain merging, (ii) enhanced absorption throughout the visible region (cf. perylene-based chromophores),^64,66,97,98^ and (iii) quantifiable prediction of π-stacking and packing density using image binarization and solvent-excluded surface models.

Our results thus extend the toolbox of Langmuir monolayer engineering from purely structural additives to multifunctional chromophoric plasticizers. This work aligns with recent efforts in colloid and interface science to develop ultrathin, functionalized membranes for selective separation and photocatalysis.^99–106^ By directly comparing experimental packing densities with theoretical cross-sectional areas, we provide a new methodology for correlating molecular structure with interfacial packing that can be generalized to other amphiphilic π-systems.

Looking ahead, we envision systematically varying molecular structures, amphiphilicity, and dipole moments to design next-generation light-harvesting membranes with tunable charge transport, catalytic activity, and long-term stability. Future work should combine direct experimental benchmarking with molecular simulations, including COSMO-RS and molecular dynamics,^107–109^ to predict miscibility, optimal loading, and aggregate structures. Such integrated approaches will accelerate the rational design of multifunctional monolayers and membranes for energy conversion, selective ion transport, and nanoscale photonic devices.

## Supplementary Material



## Data Availability

The data that support the findings of this study are available from the corresponding author upon reasonable request.

## Notes

The SI shows further data and information on UV−vis spectroscopical analysis (UV−vis spectra and Photothermal deflection spectroscopy (PDS)), AFM, Π­(mma) isotherm and BAM analysis (Π­(mma) isotherm analysis, Algorithm of the program, Average Π­(mma) isotherm analysis, Averaged Π­(mma) isotherms and BAM images, Individual Π­(mma) isotherm), Determination of space requirement for OPE molecules via aggregation, and NMR spectra (PDF)
