# Supplementary material for: A Dual-Role Amphiphilic Photosensitizer: Enhancing Structural Uniformity and Optical Properties of Langmuir Monolayers
Source: Langmuir. 2026 May 13;42(20):14013–24. doi: 10.1021/acs.langmuir.5c06004 (PMC13218362; doi:10.1021/acs.langmuir.5c06004)
Supplement: Supplementary file 1 [file la5c06004_si_001.pdf]

- Supporting Information -

A Dual-Role Amphiphilic Photosensitizer: Enhancing Structural Uniformity and Optical Properties of Langmuir Monolayers

Sarah Jasmin Finkelmeyer[a,‡], Charlotte Mankel[b,‡], Anna Elmanova[a,c,d], Konrad Hotzel[b], Zekai Ye[a], Andrea Dellith[a], Stefan Zechel[b,f,g,h], Kalina Peneva[b,e,f], Martin D. Hager[b,e,f,g,h], Ulrich S. Schubert[b,e,f,g,h], Martin Presselt[a,c,d,e,*]

[a] Leibniz Institute of Photonic Technology (IPHT), Albert-Einstein-Str. 9, 07745 Jena, Germany

[b] Institute for Organic and Macromolecular Chemistry (IOMC), Friedrich Schiller University Jena, Humboldtstr. 10, 07743 Jena, Germany

[c] Institute of Physical Chemistry, Friedrich Schiller University Jena, Helmholtzweg 4, 07743 Jena, Germany.

[d] sciclus GmbH & Co. KG, Moritz-von-Rohr-Str. 1a, 07745 Jena, Germany

[e] Center for Energy and Environmental Chemistry Jena (CEEC Jena), Friedrich Schiller University Jena, Philosophenweg 7a, 07743 Jena, Germany

[f] Jena Center for Soft Matter (JCSM), Friedrich-Schiller-University Jena, Philosophenweg 7, 07743 Jena, Germany

[g] Helmholtz Institute for Polymers in Energy Applications Jena (HIPOLE Jena), Lessingstr. 12-14, 07743, Jena, Germany

[h] Helmholtz-Zentrum Berlin für Materialien und Energie, 14109, Berlin, Germany

[‡] These authors contributed equally: Sarah Jasmin Finkelmeyer and Charlotte Mankel

*Corresponding author: martin.presselt@leibniz-ipht.de

Contents

1. UV-vis spectroscopical analysis 2
   1.1. UV-vis spectra 2
   1.2. Photothermal deflection spectroscopy (PDS) 2
2. AFM 5
3. Π(mma) isotherm and BAM analysis 5
   3.1. Π(mma) isotherm analysis 5
   3.2. Algorithm of the program 5
   3.3. Average Π(mma) isotherm analysis 6
   3.4. Averaged Π(mma) isotherms and BAM images 7
   3.5. Individual Π(mma) isotherm 18
4. Determination of space requirement for OPE molecules via aggregation 29
5. NMR spectra 34
6. References 35

1. UV-vis spectroscopical analysis

1.1. UV-vis spectra

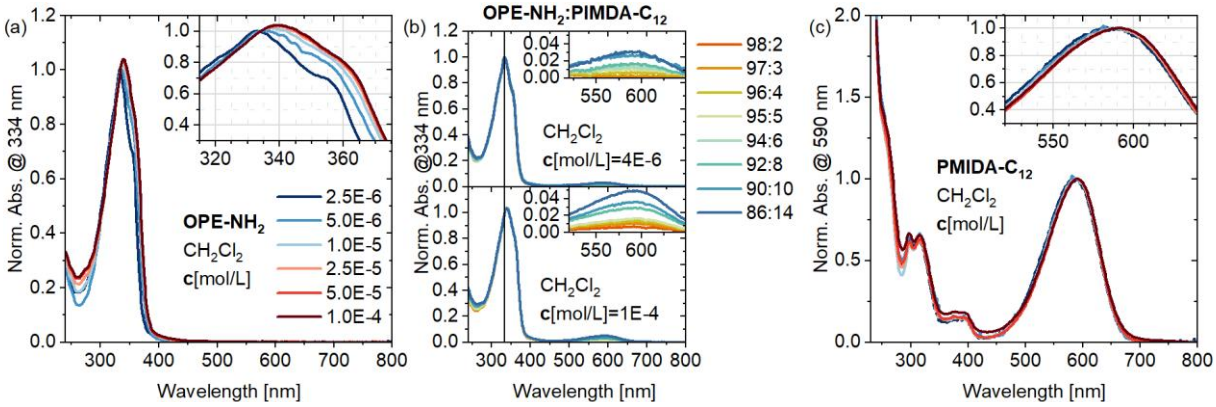

Figure S1: UV-vis absorption spectra of (a) ***OPE-NH₂*** *in different concentrations, (b) mixtures of* ***OPE-NH₂ : PMIDA-C₁₂*** *(upper panel) low concentration and (lower panel) high concentration, and (c****) PMIDA-C₁₂*** *in different concentrations (same as listed in panel (a)). Please note that in lower panel of (b) absorption is not normalized to the maximum but at the same wavelength as the mixtures of the lower concentration (upper panel, (b) therefore it seems that the* ***PMIDA-C₁₂*** *absorption is more intense in the higher concentration (panel (b) insets).*

1.2. Photothermal deflection spectroscopy (PDS)

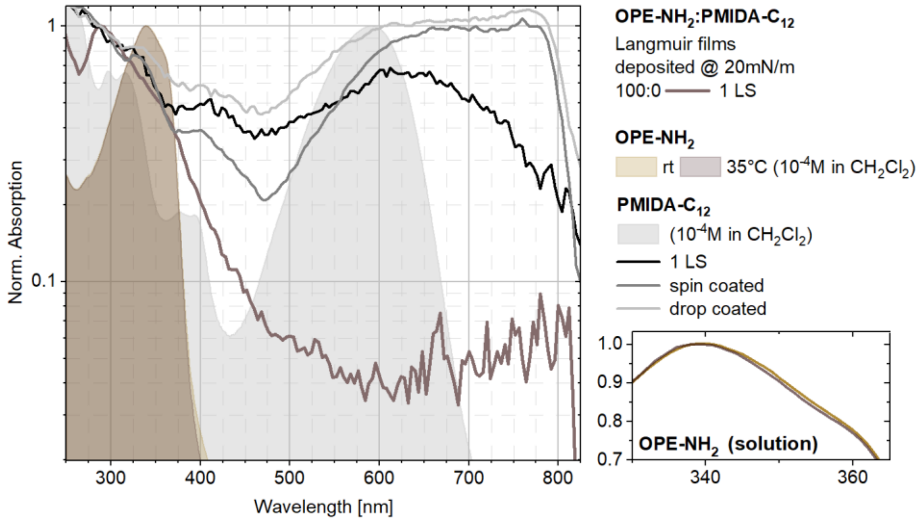

Figure S2:PDS and UV-vis absorption spectra, not smoothed data, highlighting ***OPE-NH₂*** *absorption properties.*

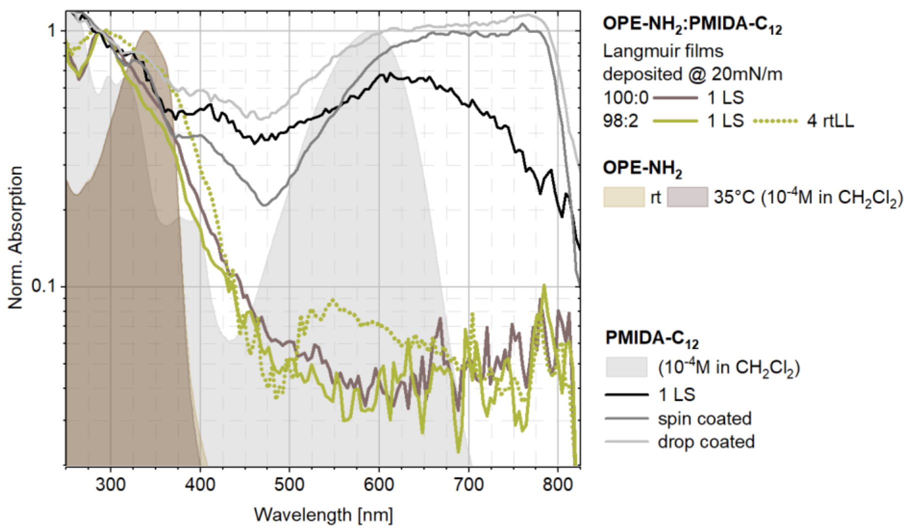

*Figure S3:PDS and UV-vis absorption spectra, not smoothed data, highlighting **OPE-NH$_2$: PMIDA-C$_{12}$** molar mixing of **98:2** absorption properties.*

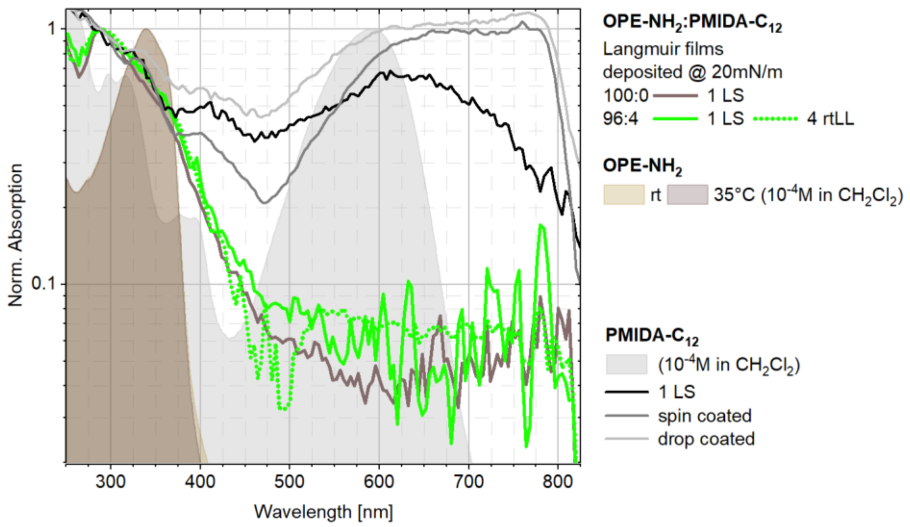

*Figure S4:PDS and UV-vis absorption spectra, not smoothed data, highlighting **OPE-NH$_2$: PMIDA-C$_{12}$** molar mixing of **96:4** absorption properties.*

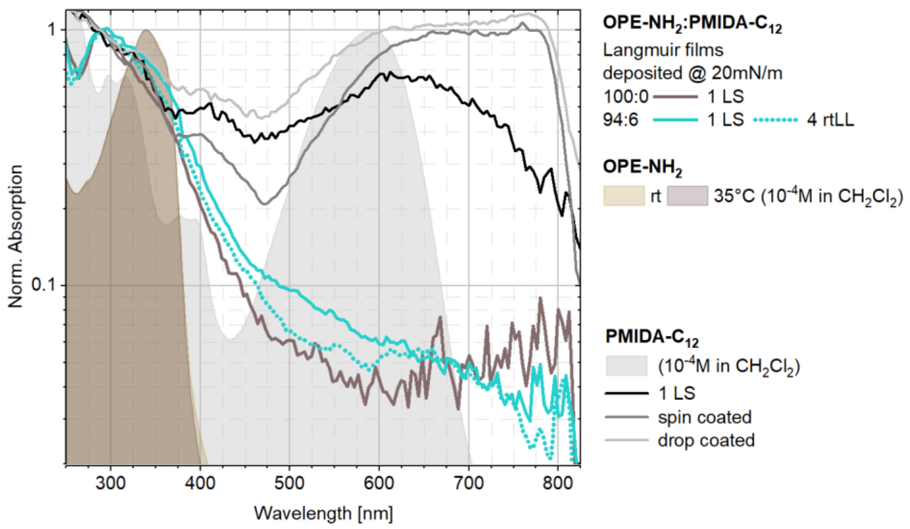

*Figure S5:PDS and UV-vis absorption spectra, not smoothed data, highlighting **OPE-NH$_2$: PMIDA-C$_{12}$** molar mixing of **94:6** absorption properties.*

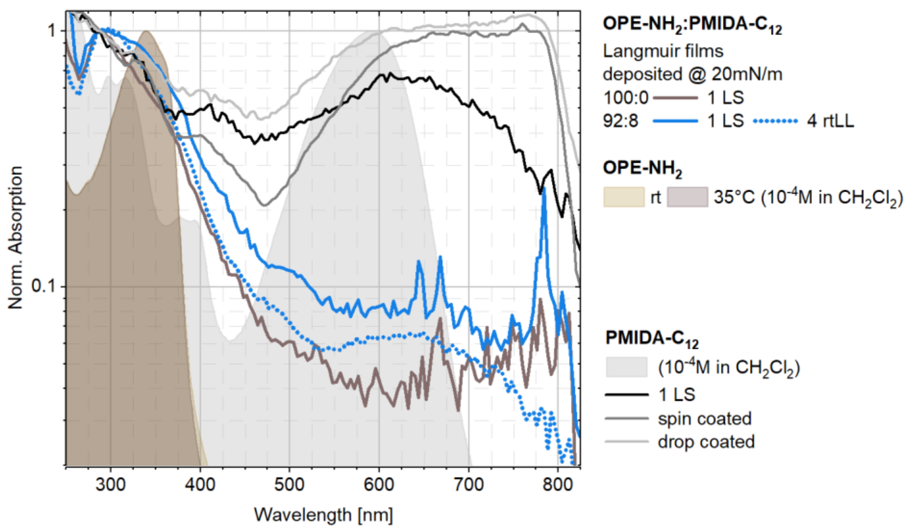

*Figure S6:PDS and UV-vis absorption spectra, not smoothed data, highlighting **OPE-NH$_2$: PMIDA-C$_{12}$** molar mixing of **92:8** absorption properties.*

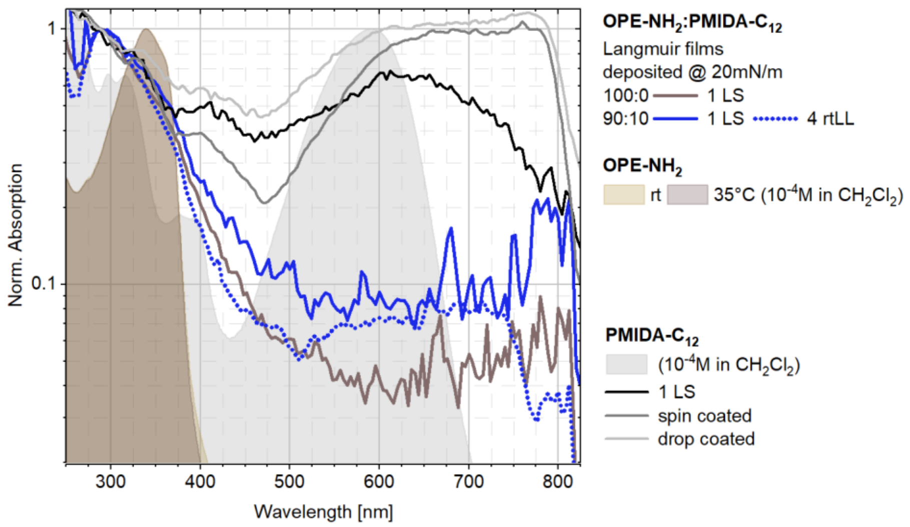

*Figure S7:PDS and UV-vis absorption spectra, not smoothed data, highlighting **OPE-NH$_2$: PMIDA-C$_{12}$** molar mixing of **90:10** absorption properties.*

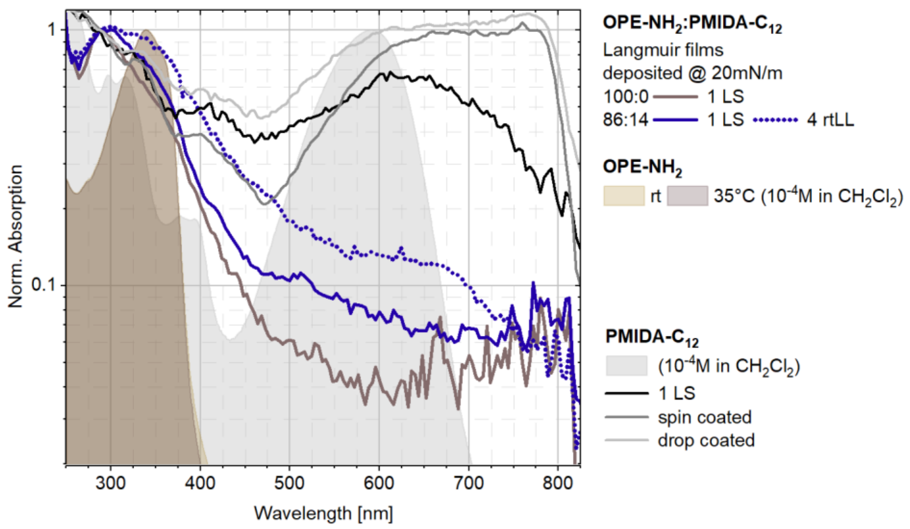

*Figure S8:PDS and UV-vis absorption spectra, not smoothed data, highlighting **OPE-NH$_2$: PMIDA-C$_{12}$** molar mixing of **86:14** absorption properties.*

2. AFM

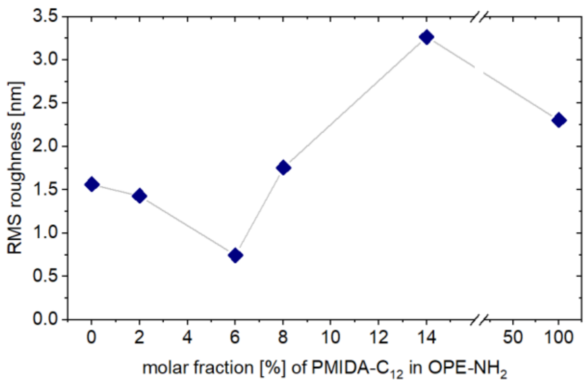

Figure S9: RMS roughness of ***OPE-NH₂ : PMIDA-C₁₂*** *Langmuir Schaefer films on silicon substrates derived from 100 µm AFM scan.*

3. Π(mma) isotherm and BAM analysis

3.1. Π(mma) isotherm analysis

For Π(mma) isotherm data analysis the JuPytR notebooks[2, 3] were utilized, namely using Python3[4] within Anaconda environment. The program takes raw data from Π(mma) isotherm measurements, processes each file separately, and then averages the data and uncertainties for each experiment's Π(mma) isotherms.

The surface compression modulus C_S^{-1} is calculated using the analytical first derivative of the averaged interpolation functions of the experimentally obtained Π(mma) isotherms:[5]

$$C_S{}^{-1} = -\Pi \frac{d\Pi}{dmma}. \qquad (S1)$$

Gaussian function is taken as the sum of Gaussian peak with an additional linear function:[6]

$$f(x) = m \cdot (x - x_c) + \frac{A}{w\sqrt{\frac{\pi}{2}}} exp\left(-\frac{x-x_c}{w}\right)^2. \qquad (S2)$$

The function takes an input *x* and parameters: *m*, which is the linear coefficient that scales the linear component of the model. x_c is the x-coordinate of the centre of the Gaussian peak. *A* is the amplitude of the Gaussian peak, determining its magnitude. *w* is the standard deviation of the Gaussian peak, controlling the spread of the peak.

3.2. Algorithm of the program

The input file specifies boundaries for fitting parameters, the fitting range, initial guesses, and points of interest (points which shall be highlighted on the averaged Π(mma) isotherm). The data within the fitting range is fitted using a Gaussian function, with initial guesses and boundary constraints from the input file.

Optimization is done using the Levenberg-Marquardt algorithm to minimize the differences between model predictions and observed data points.[7-13] Standard deviations are calculated using the same optimization function. After fitting, the function returns optimal parameter values and a covariance matrix,[14] which shows the estimated covariance between fitted parameters. The variances of the fitted parameters are found on the diagonal of the covariance matrix.

The uncertainty of the mean molecular area at maximum of surface compression modulus (mma_c) is taken as approximately equal to the uncertainty of x_c. The position of mma_c is determined by maximum of the fitting function. Therefore:[6]

$$mma_C Err = \sqrt{xcErr^2 + mma_C meanErr^2} \qquad (S3)$$

But as no mean error is presented for a single Π(mma) isotherm, then one takes:

$$mma_C Err \approx xcErr. \quad \text{(S4)}$$

The corresponding surface pressure Π_C is also reported. The program then performs linear fitting within a specified range on the isotherm part of the subplot and calculates corresponding uncertainties.

For linear extrapolation of the Π(mma) isotherm narrow data ranges whose mean molecular areas correspond to the top of the compression modulus peaks are used. The fitting range for linear fit is taken as w/3 around the position of the maximum point mma_c to obtain extrapolated mean molecular areas mma_0 (also referred to as A_0 in the figures in the SI).

3.3. Average Π(mma) isotherm analysis

Since the mean molecular area points may vary between different Π(mma) isotherms, the data files are first extrapolated to cover the range from the minimum possible mean molecular area to the maximum among the Π(mma) isotherms being averaged. A uniform x-axis is introduced for this extrapolation. After extrapolation, the surface pressure values are averaged across different data files. The resulting average Π(mma) isotherm is then used for all subsequent procedures described earlier. The program saves the resulting columns into a .csv file, including mean molecular area, average surface pressure, mean molecular area error, surface compression modulus, and Gaussian function.

To calculate averages for specific points of the Π(mma) isotherms, the values are extracted from the outputs of the individual Π(mma) isotherms. These values are then averaged, and for uncertainties, a specific formula is applied:

$$\Delta\bar{x} = \frac{\sigma}{\sqrt{n}} \quad \text{(S5)}$$

Where *n* is the number of different files (Π(mma) isotherms), for which the specific value was calculated and σ is the standard deviation. Then for each of the values on average Π(mma) isotherm one has the value:

$$\bar{x} \pm \Delta\bar{x} \quad \text{(S6)}$$

Where $\bar{x}$ is the average of the value. This is done for mma_c, $C_{s,max}^{-1}$, Π_C and mma_0 (also referred to as A_0 in the figures in the SI), which are, correspondingly, mean molecular area at maximum surface compression modulus, maximum of surface compression modulus, pressure at maximum surface compression modulus and the point of intersection of the extrapolated linear approximation function, derived from interpolation of the Π(mma) isotherm data points in the w/3 range, with the mean molecular area axis (x-axis).

3.4. Averaged Π(mma) isotherms and BAM images

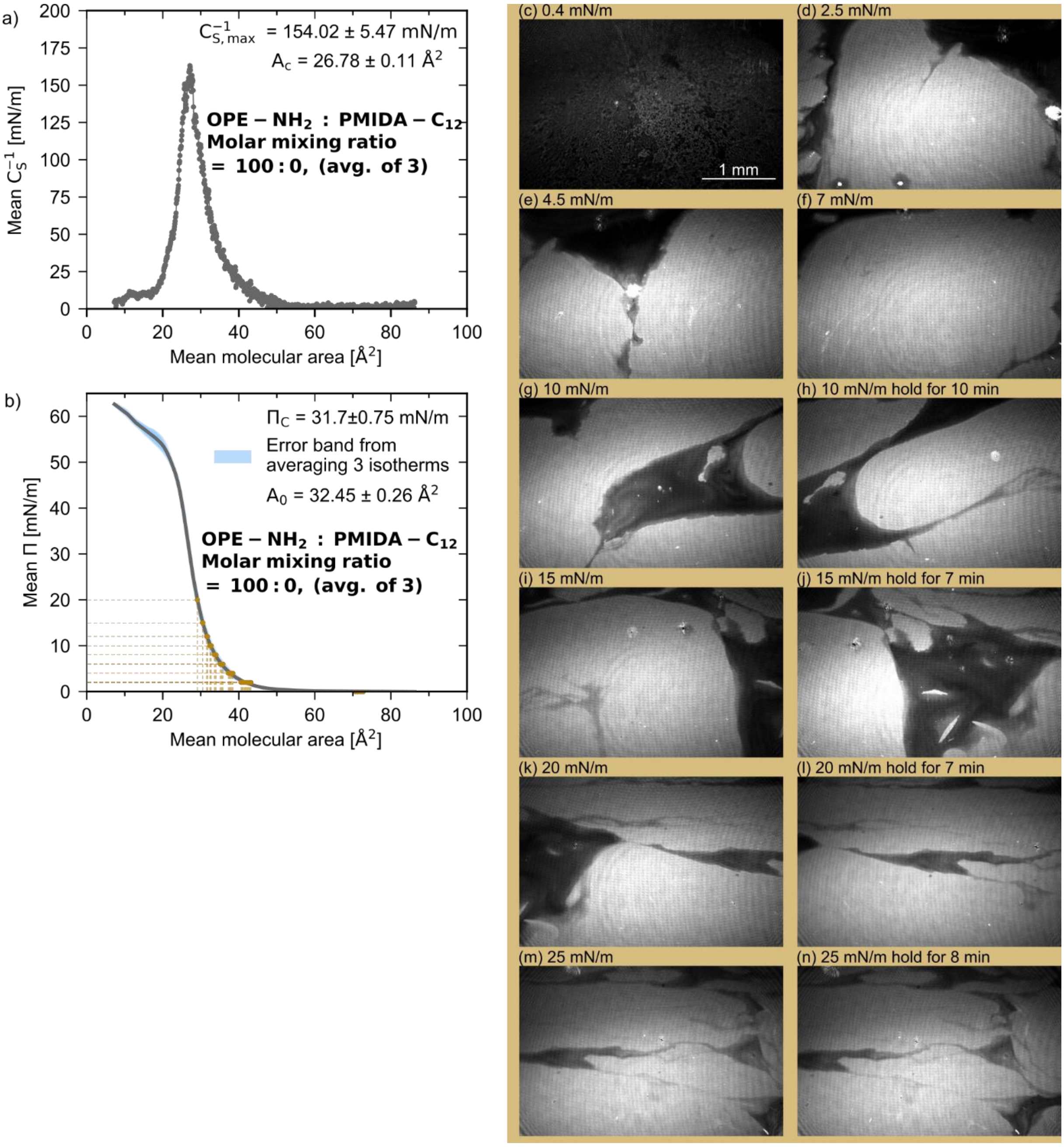

Figure S10: Π(mma) isotherm analysis of ***OPE-NH$_2$*** *:* ***PMIDA-C$_{12}$*** *mixture with molar mixing ratio [%]* ***100:0*** *in (a) and (b). (c) to (j) BAM images of the course of the Π(mma) isotherm up to 25 mN/m. Reproduced and slightly adapted with permission from Finkelmeyer et al.[1] Copyright 2025 Journal of Colloid and Interface Science (Elsevier).*

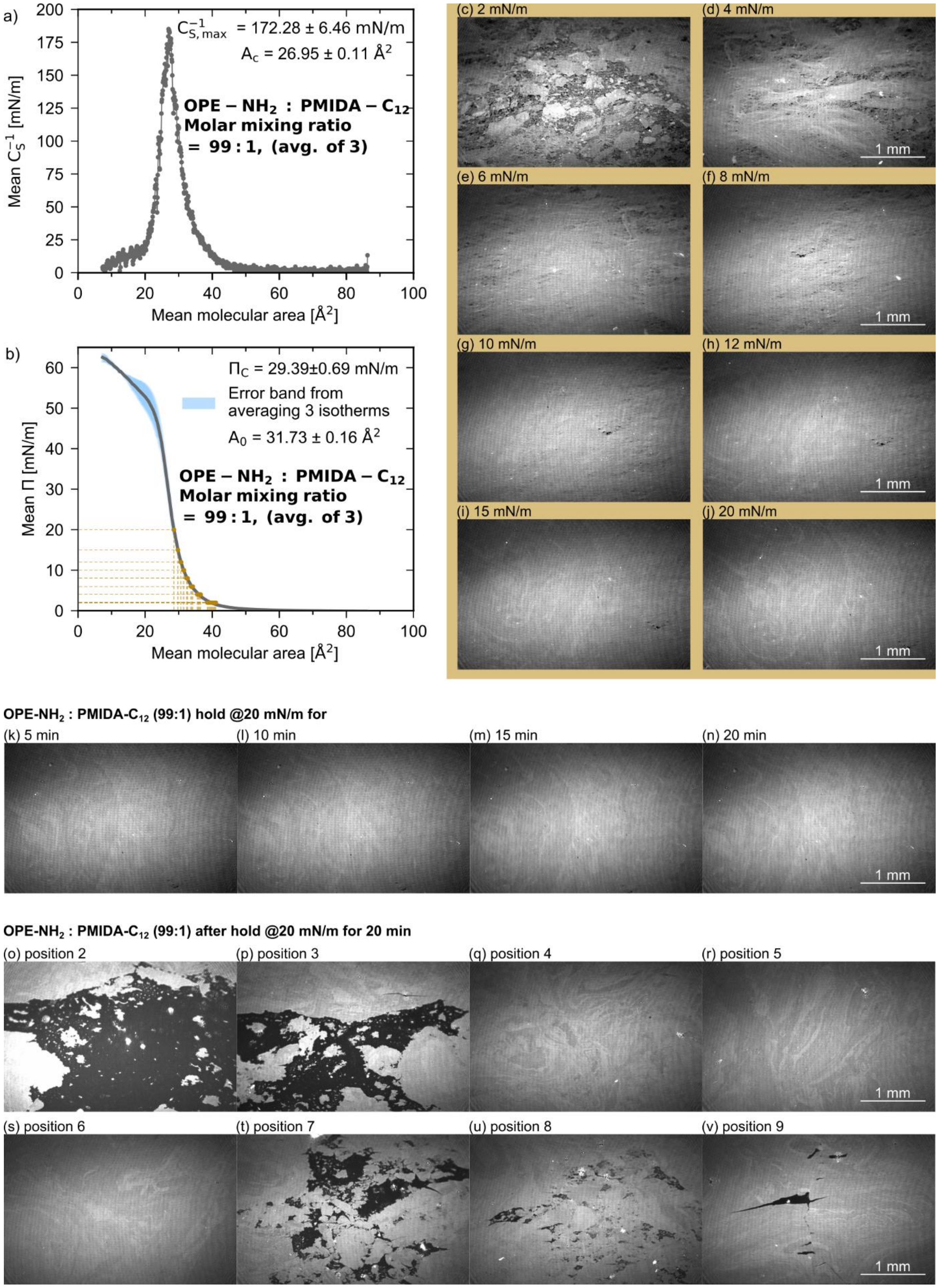

*Figure S11: Π(mma) isotherm analysis of **OPE-NH$_2$** : **PMIDA-C$_{12}$** mixture with molar mixing ratio [%] **99:1** in (a) and (b). (c) to (j) BAM images of the course of the Π(mma) isotherm up to 20 mN/m. (k) to (n) hold at 20 mN/m for different time duration. (o) to (v) different spots of the LL after 20 min hold at 20 mN/m.*

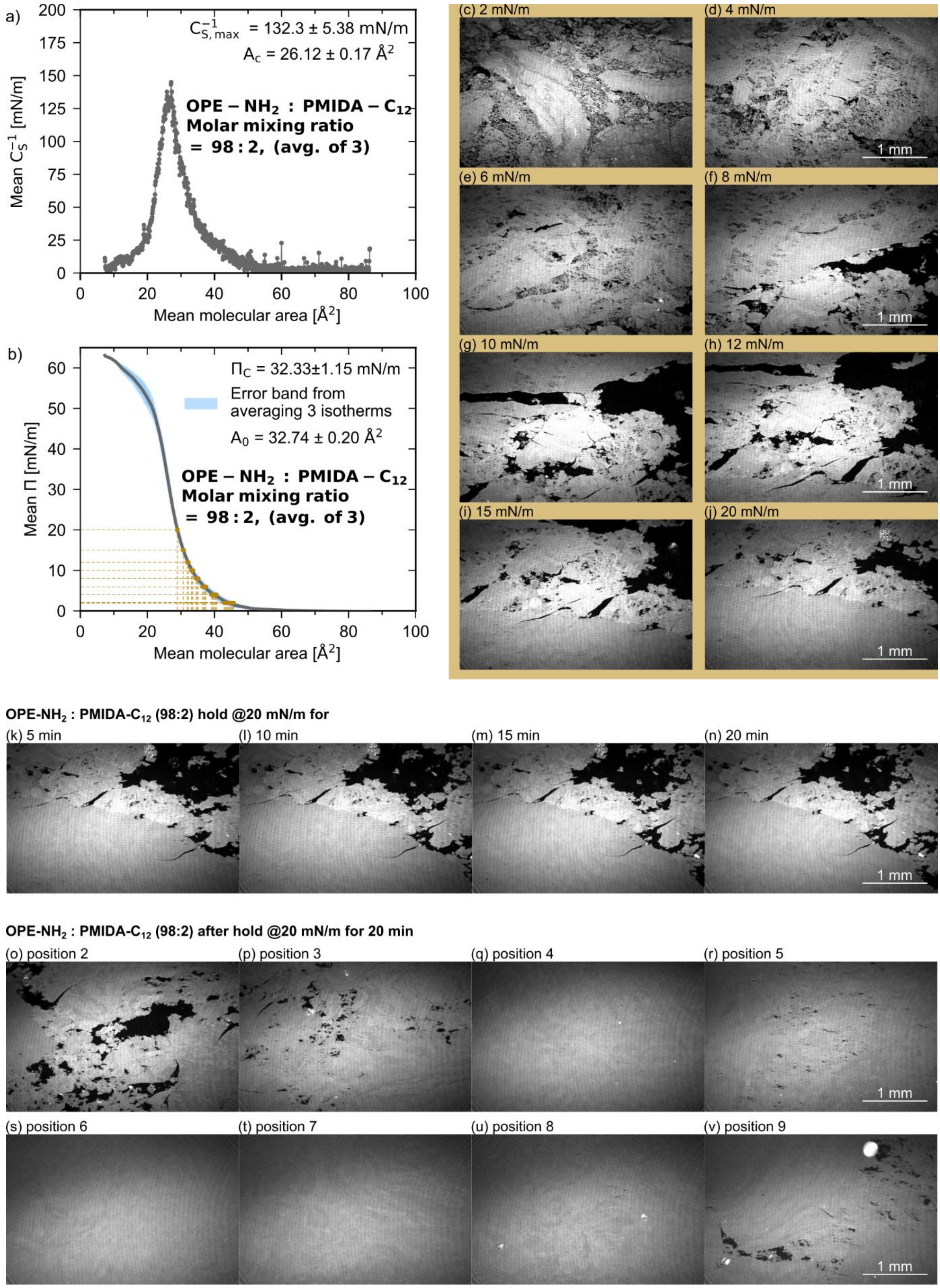

*Figure S12: Π(mma) isotherm analysis of **OPE-NH$_2$: PMIDA-C$_{12}$** mixture with molar mixing ratio [%] **98:2** in (a) and (b). (c) to (j) BAM images of the course of the Π(mma) isotherm up to 20 mN/m. (k) to (n) hold at 20 mN/m for different time duration. (o) to (v) different spots of the LL after 20 min hold at 20 mN/m.*

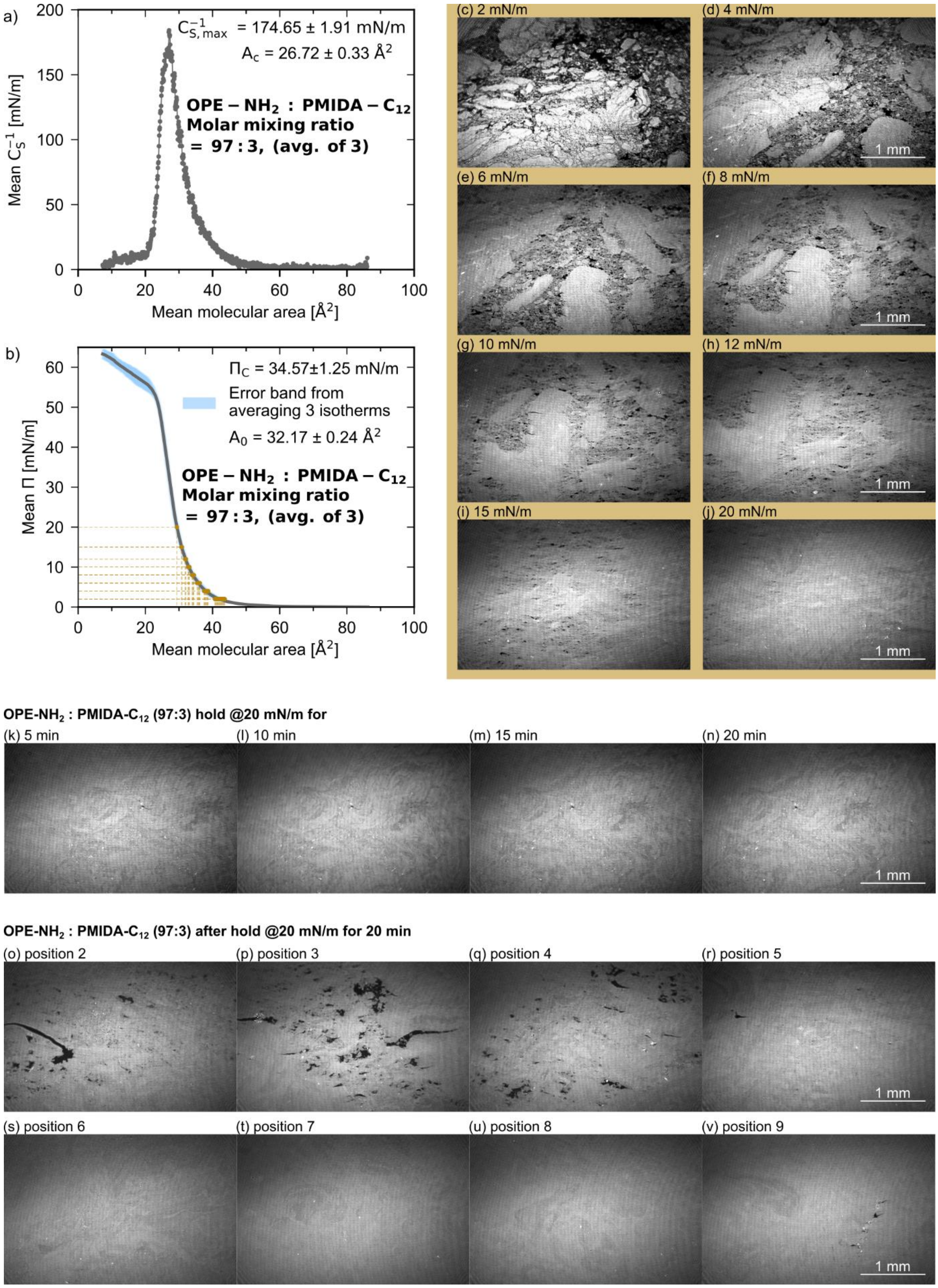

*Figure S13: Π(mma) isotherm analysis of **OPE-NH$_2$: PMIDA-C$_{12}$** mixture with molar mixing ratio [%] **97:3** in (a) and (b). (c) to (j) BAM images of the course of the Π(mma) isotherm up to 20 mN/m. (k) to (n) hold at 20 mN/m for different time duration. (o) to (v) different spots of the LL after 20 min hold at 20 mN/m.*

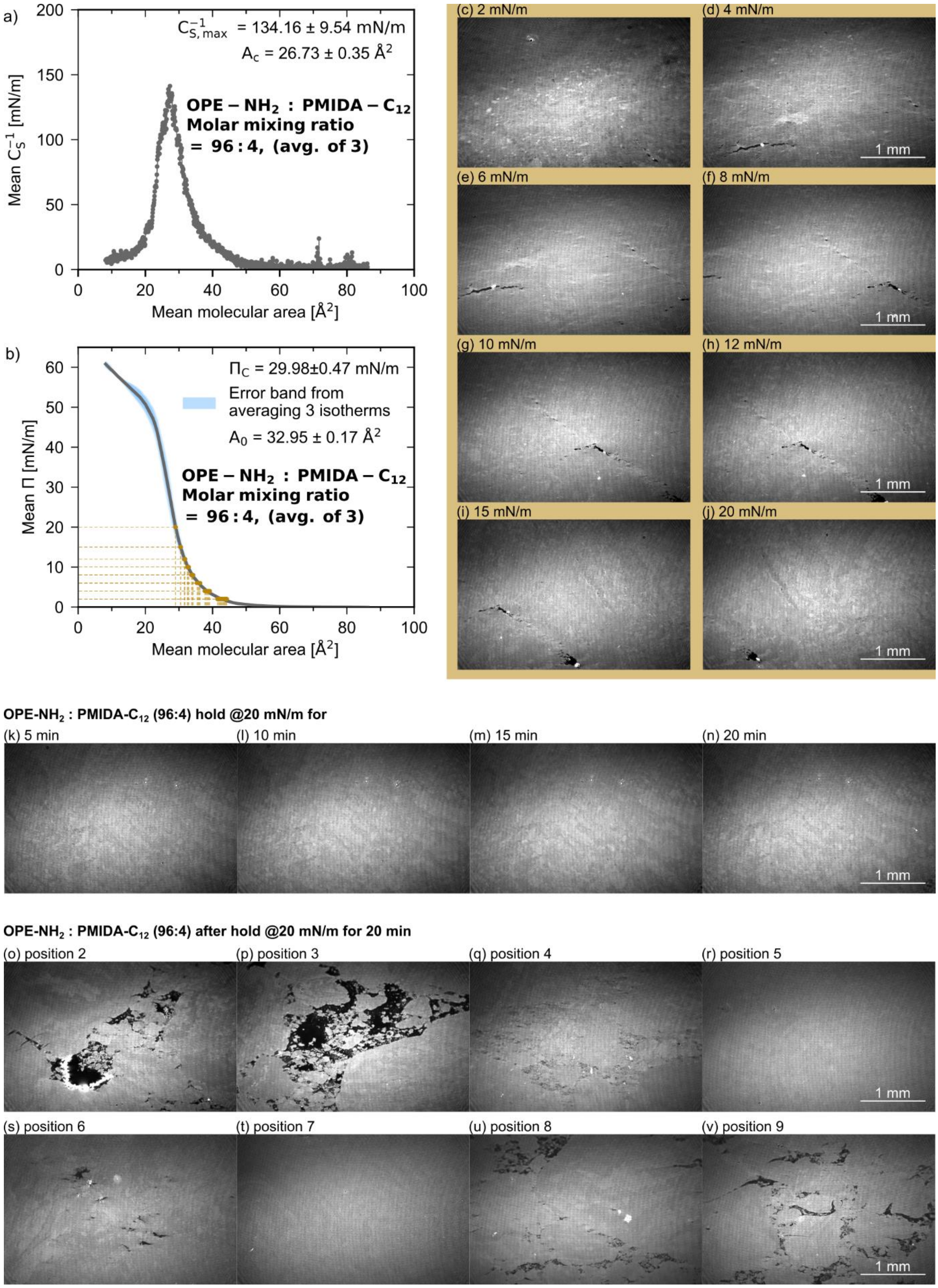

*Figure S14: Π(mma) isotherm analysis of **OPE-NH$_2$: PMIDA-C$_{12}$** mixture with molar mixing ratio [%] **96:4** in (a) and (b). (c) to (j) BAM images of the course of the Π(mma) isotherm up to 20 mN/m. (k) to (n) hold at 20 mN/m for different time duration. (o) to (v) different spots of the LL after 20 min hold at 20 mN/m.*

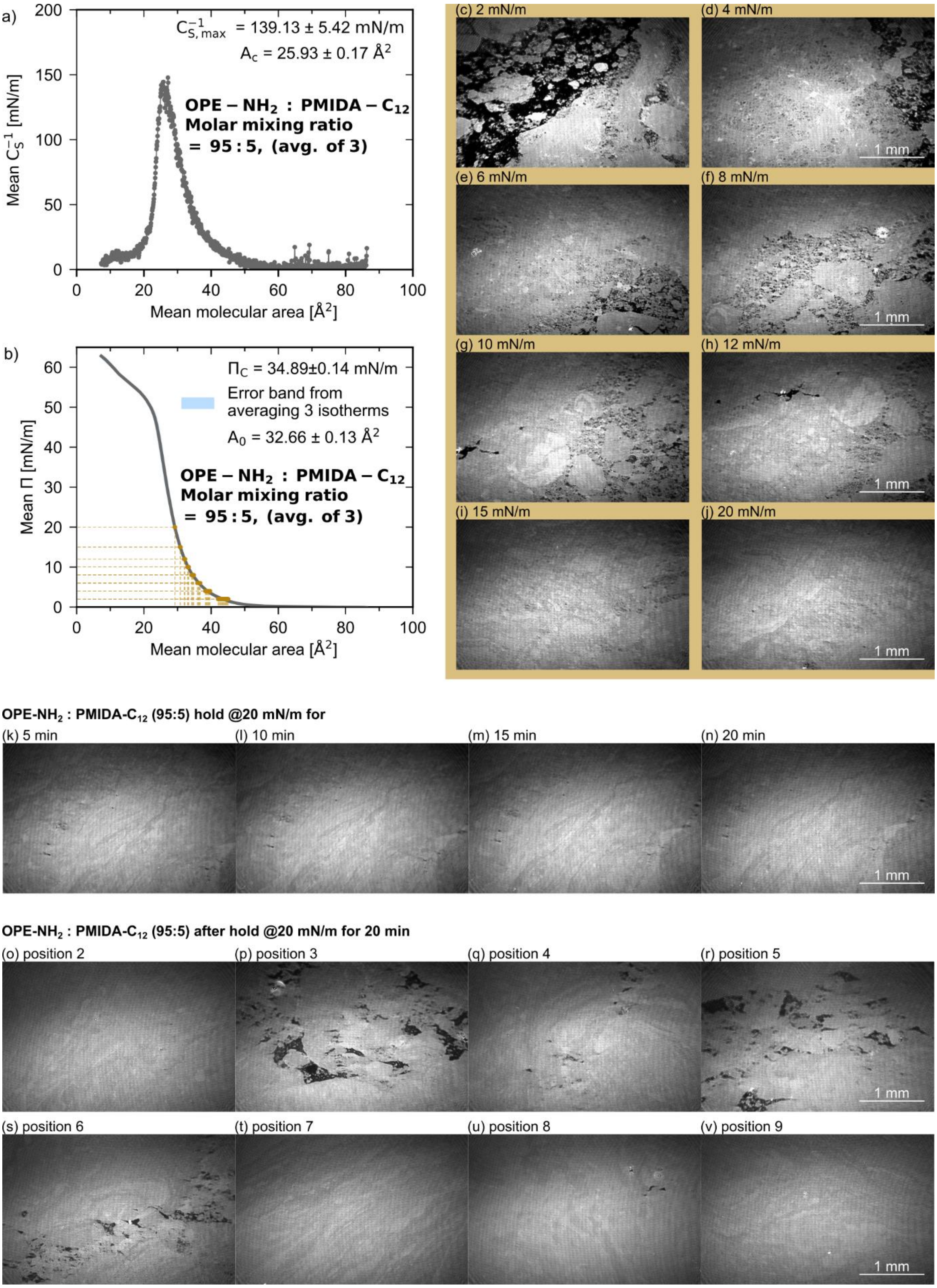

Figure S15: Π(mma) isotherm analysis of ***OPE-NH$_2$: PMIDA-C$_{12}$*** *mixture with molar mixing ratio [%]* ***95:5*** *in (a) and (b). (c) to (j) BAM images of the course of the Π(mma) isotherm up to 20 mN/m. (k) to (n) hold at 20 mN/m for different time duration. (o) to (v) different spots of the LL after 20 min hold at 20 mN/m.*

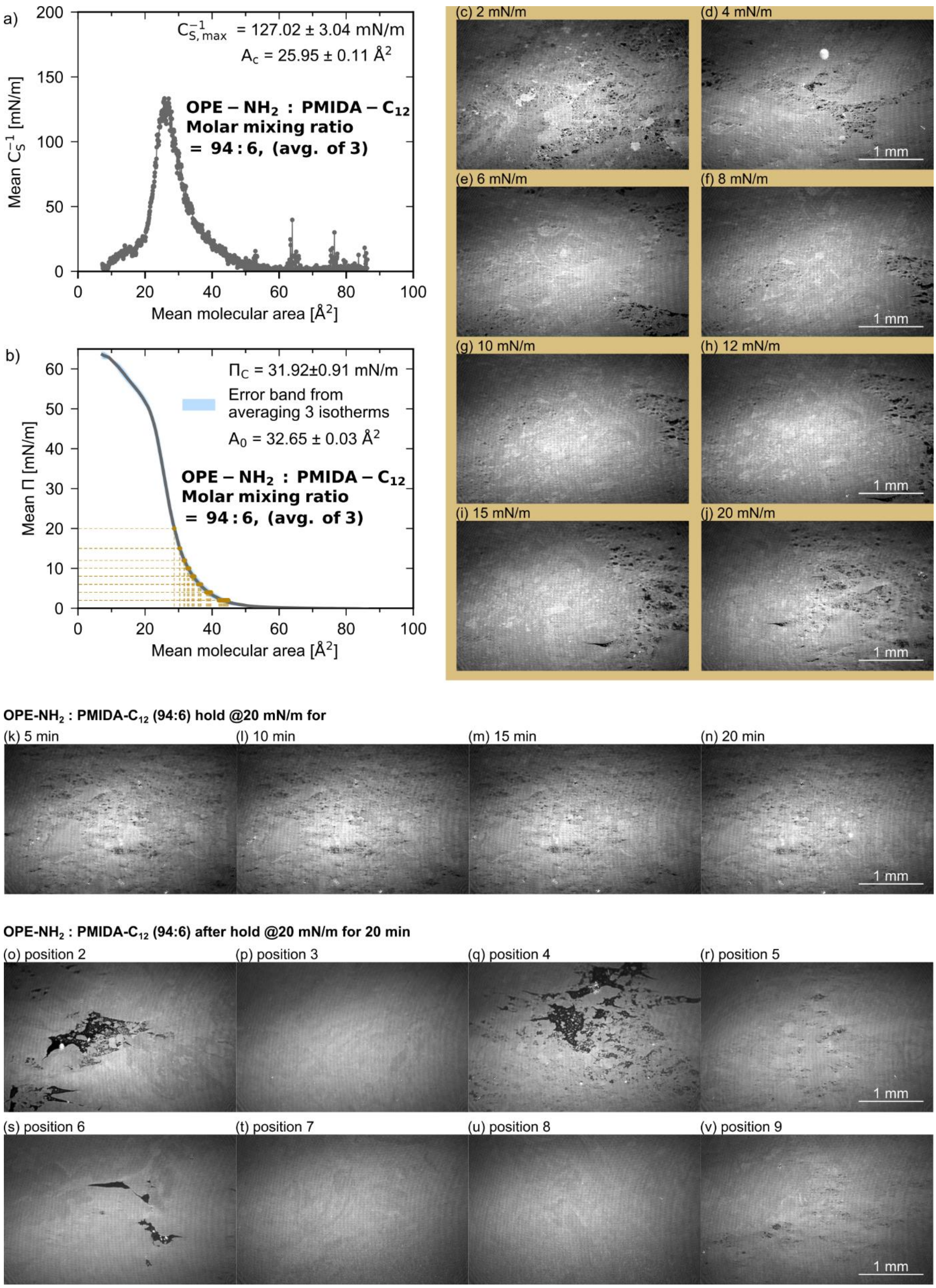

Figure S16: Π(mma) isotherm analysis of ***OPE-NH$_2$: PMIDA-C$_{12}$*** *mixture with molar mixing ratio [%]* ***94:6*** *in (a) and (b). (c) to (j) BAM images of the course of the Π(mma) isotherm up to 20 mN/m. (k) to (n) hold at 20 mN/m for different time duration. (o) to (v) different spots of the LL after 20 min hold at 20 mN/m.*

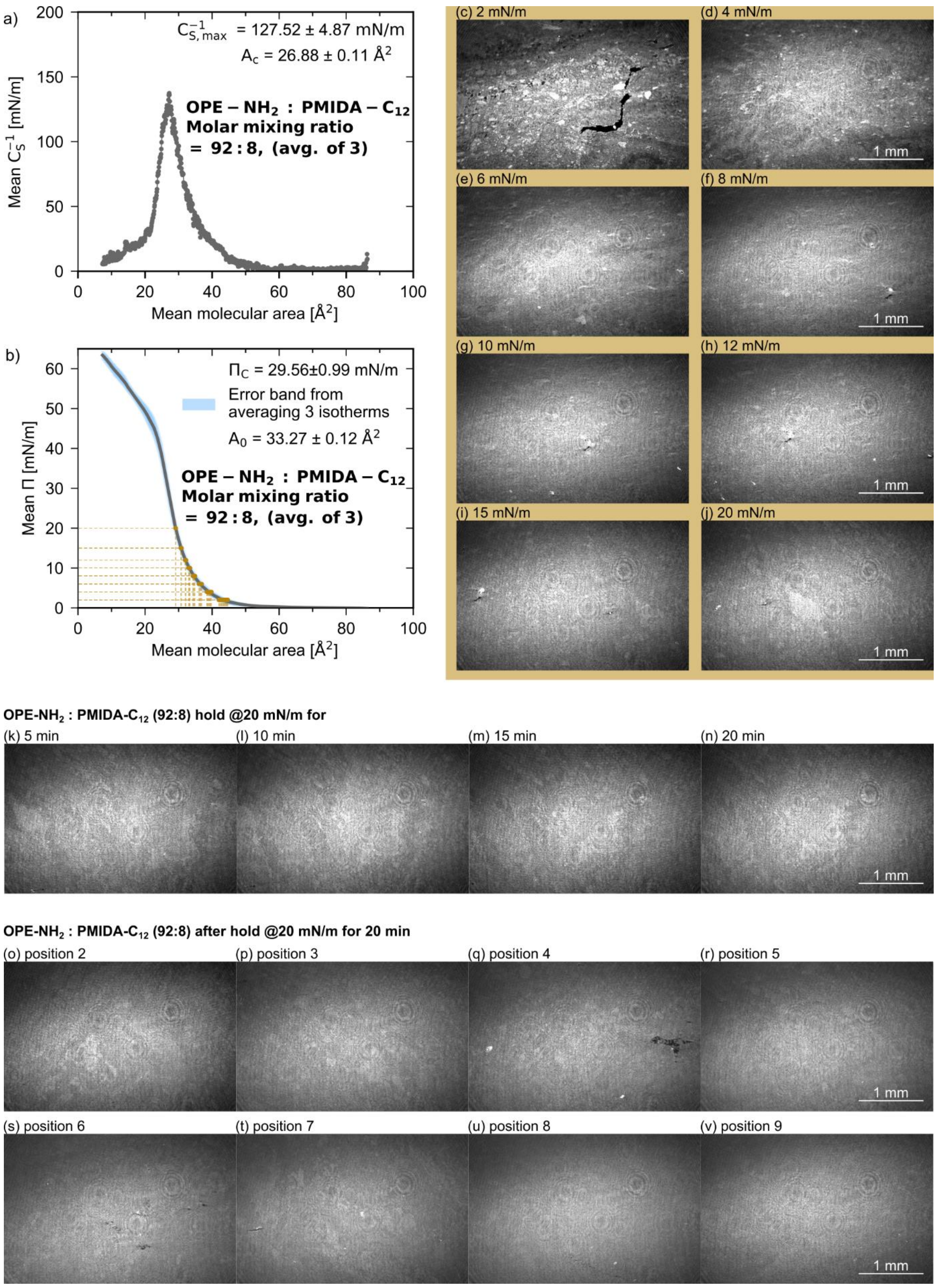

*Figure S17: Π(mma) isotherm analysis of **OPE-NH$_2$: PMIDA-C$_{12}$** mixture with molar mixing ratio [%] **92:8** in (a) and (b). (c) to (j) BAM images of the course of the Π(mma) isotherm up to 20 mN/m. (k) to (n) hold at 20 mN/m for different time duration. (o) to (v) different spots of the LL after 20 min hold at 20 mN/m.*

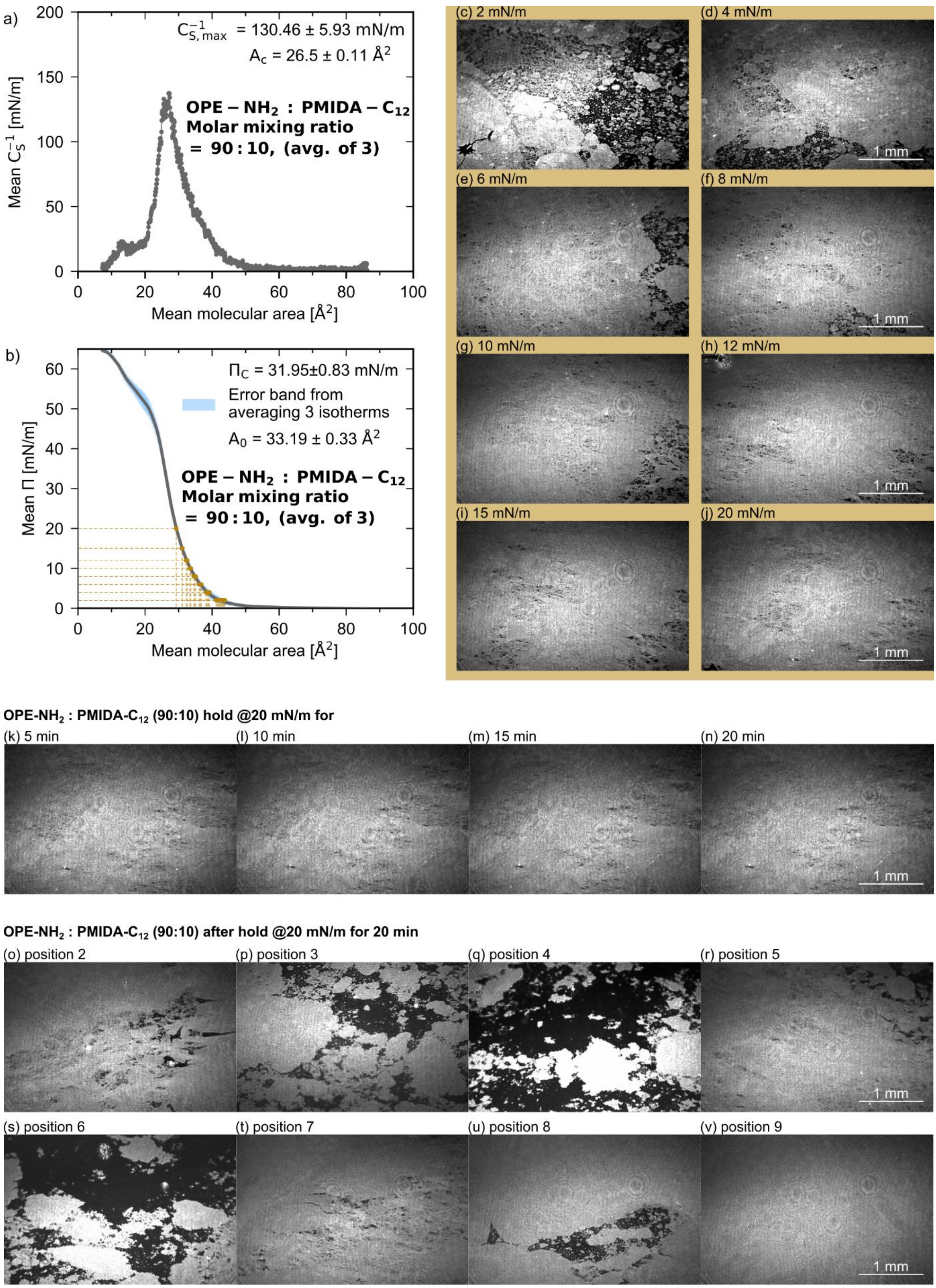

*Figure S18: Π(mma) isotherm analysis of **OPE-NH$_2$** : **PMIDA-C$_{12}$** mixture with molar mixing ratio [%] **90:10** in (a) and (b). (c) to (j) BAM images of the course of the Π(mma) isotherm up to 20 mN/m. (k) to (n) hold at 20 mN/m for different time duration. (o) to (v) different spots of the LL after 20 min hold at 20 mN/m.*

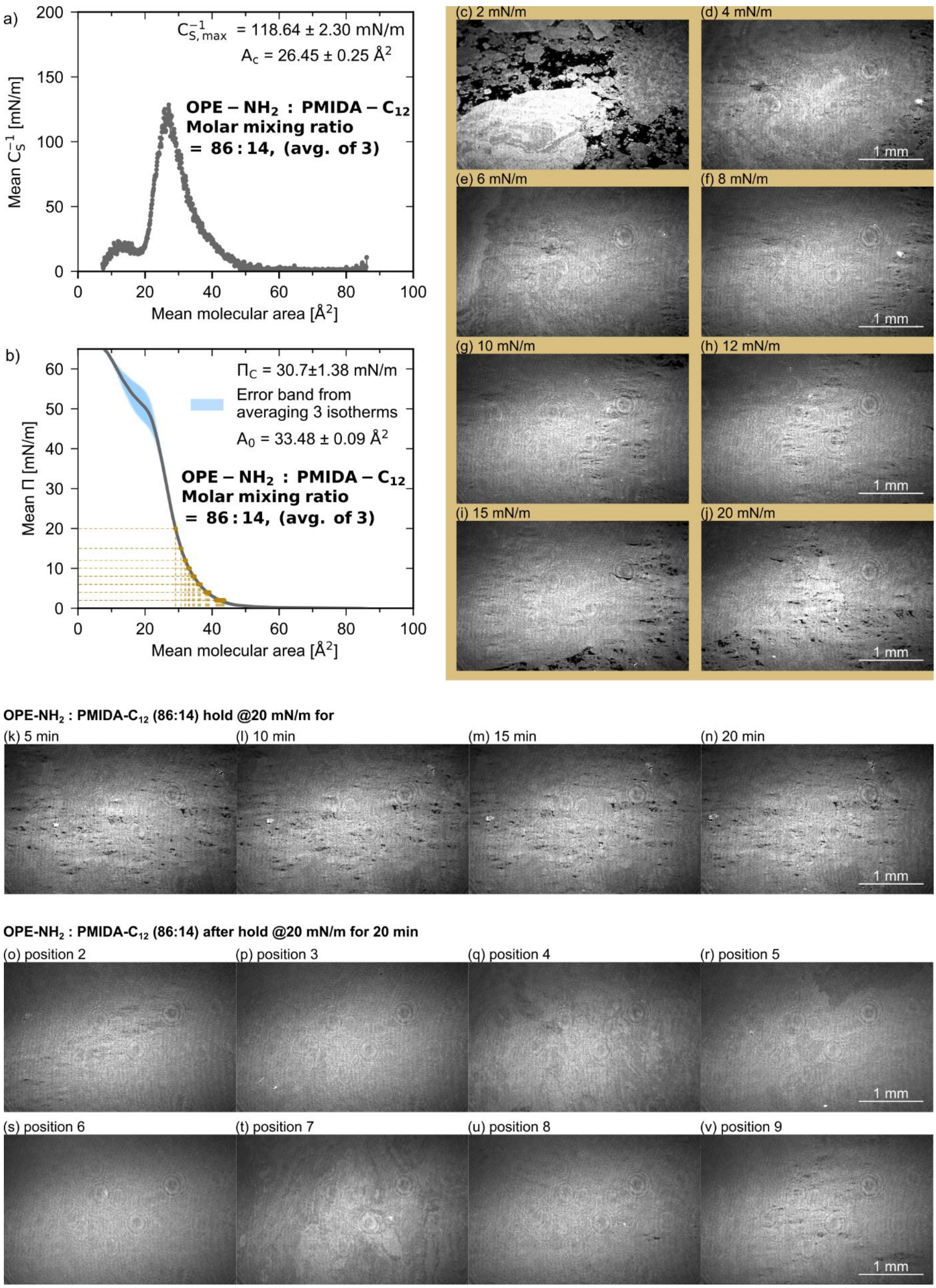

*Figure S19: Π(mma) isotherm analysis of **OPE-NH$_2$** : **PMIDA-C$_{12}$** mixture with molar mixing ratio [%] **86:14** in (a) and (b). (c) to (j) BAM images of the course of the Π(mma) isotherm up to 20 mN/m. (k) to (n) hold at 20 mN/m for different time duration. (o) to (v) different spots of the LL after 20 min hold at 20 mN/m.*

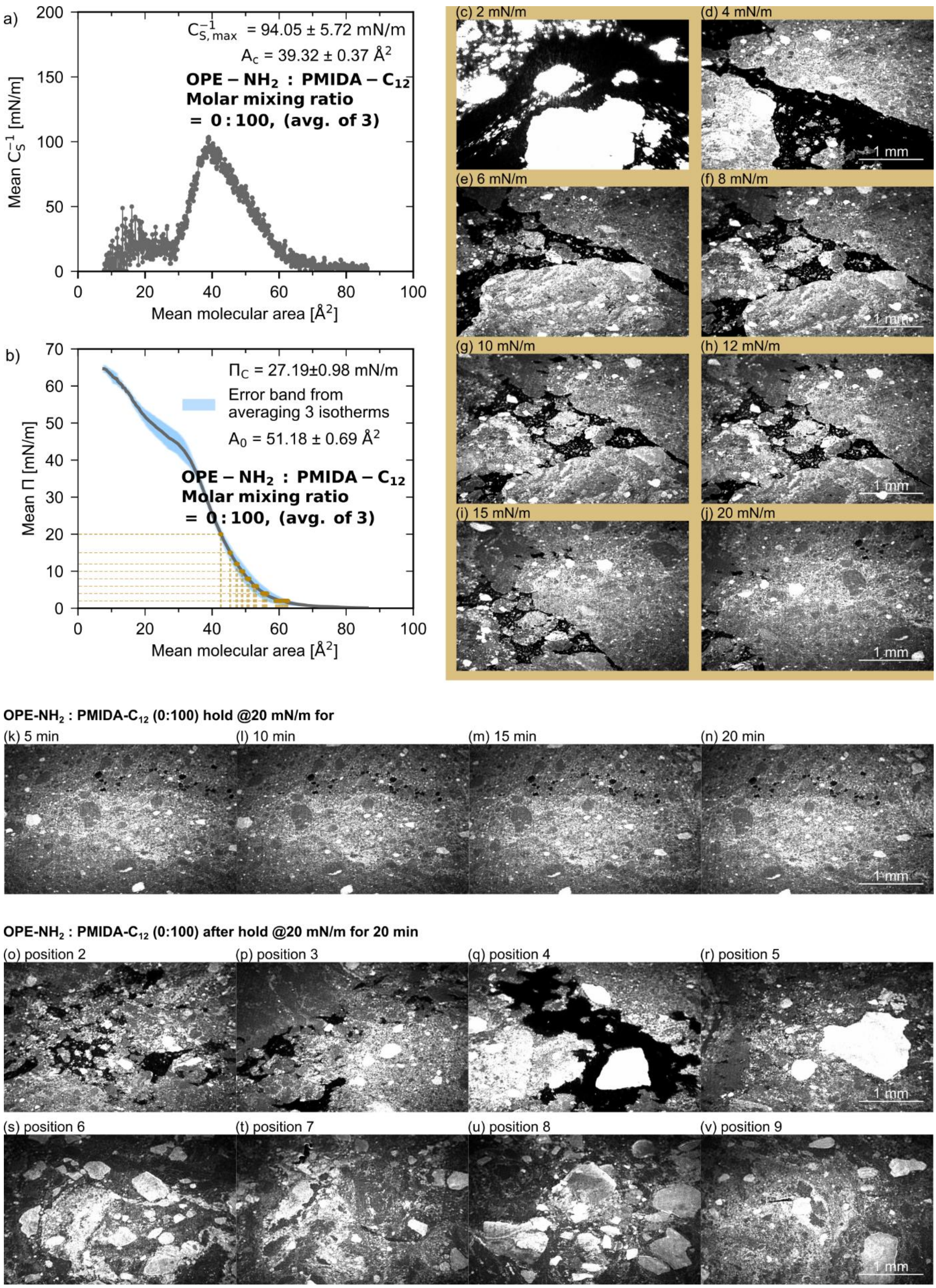

*Figure S20: Π(mma) isotherm analysis of pristine **PMIDA-C$_{12}$** in (a) and (b). (c) to (j) BAM images of the course of the Π(mma) isotherm up to 20 mN/m. (k) to (n) hold at 20 mN/m for different time duration. (o) to (v) different spots of the LL after 20 min hold at 20 mN/m.*

3.5. Individual Π(mma) isotherm

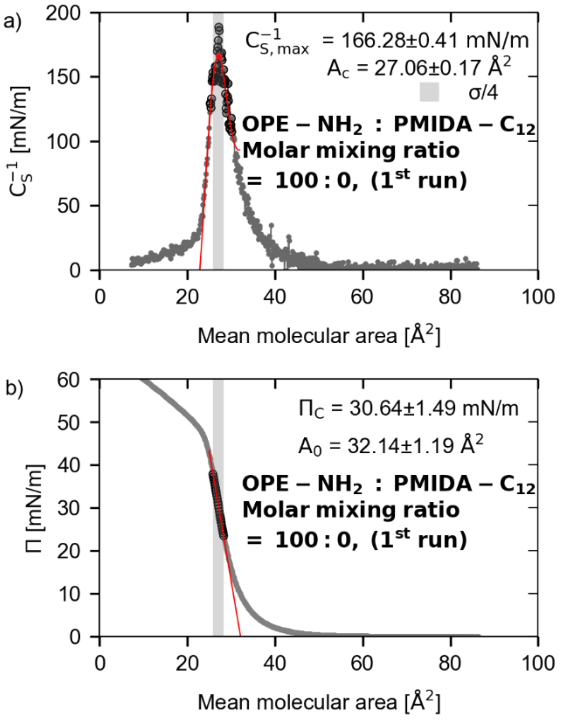

Figure S21: Analysis of first individual Π(mma) isotherm of pristine OPE-NH$_2$.

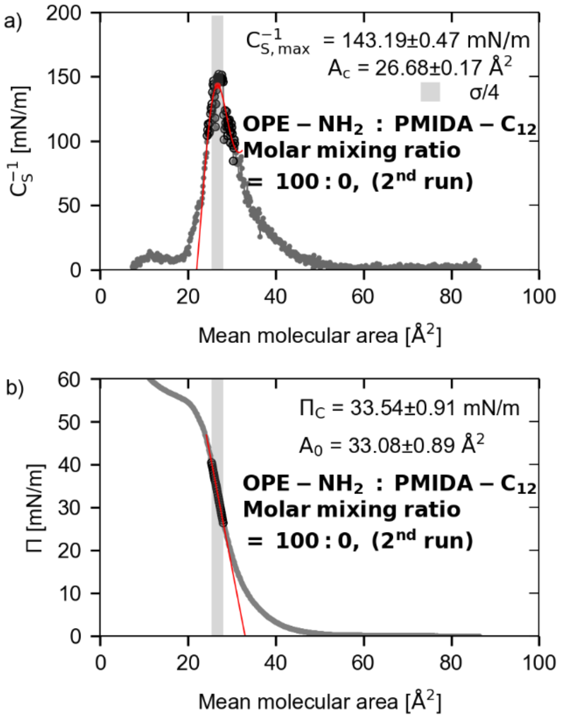

Figure S22: Analysis of second individual Π(mma) isotherm of pristine OPE-NH$_2$.

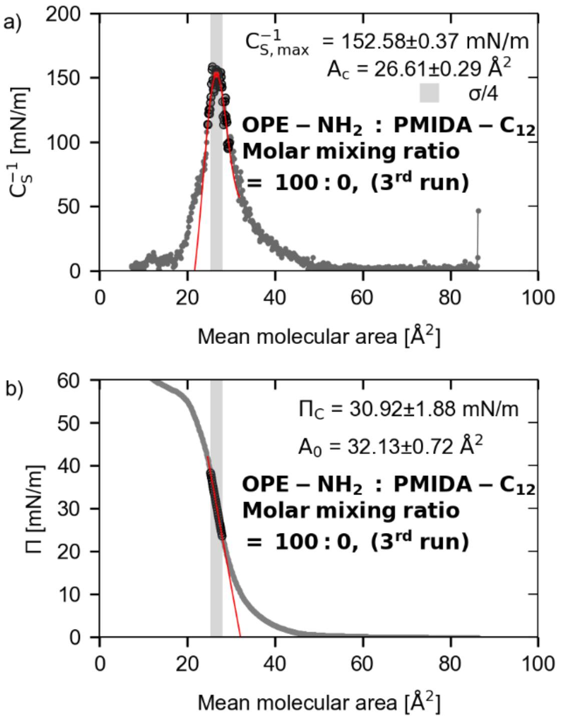

Figure S23: Analysis of third individual Π(mma) isotherm of pristine OPE-NH$_2$.

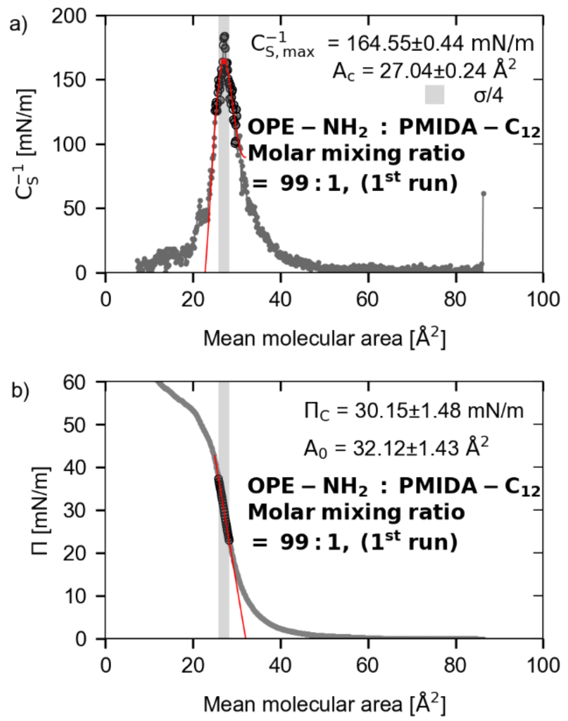

Figure S24: Analysis of first individual Π(mma) isotherm of OPE-NH_2 : PMIDA-C_{12} mixture with molar mixing ratio [%] 99:1.

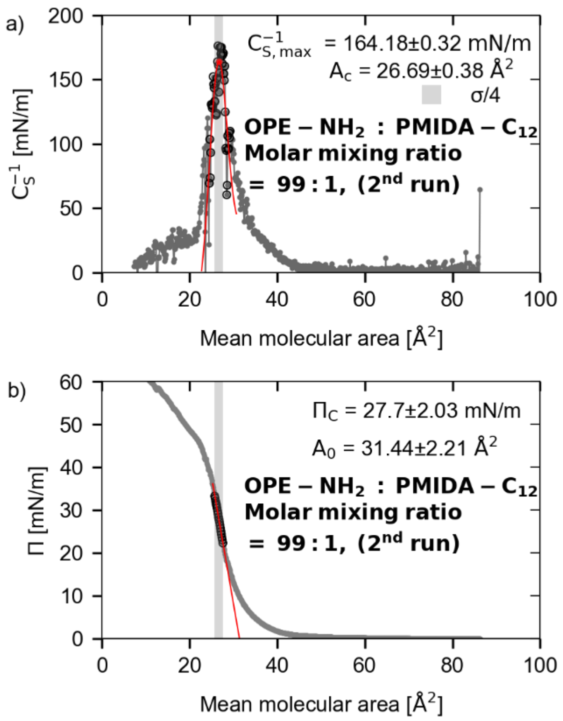

Figure S25: Analysis of second individual Π(mma) isotherm of OPE-NH_2 : PMIDA-C_{12} mixture with molar mixing ratio [%] 99:1.

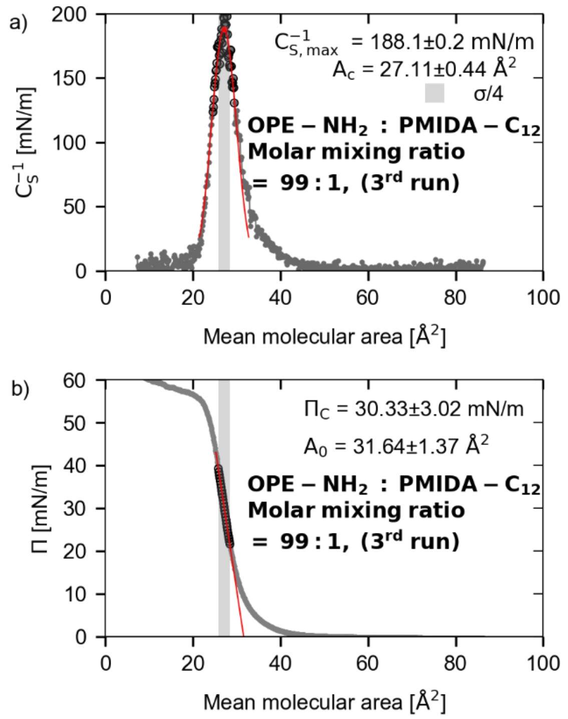

Figure S26: Analysis of third individual Π(mma) isotherm of OPE-NH_2 : PMIDA-C_{12} mixture with molar mixing ratio [%] 99:1.

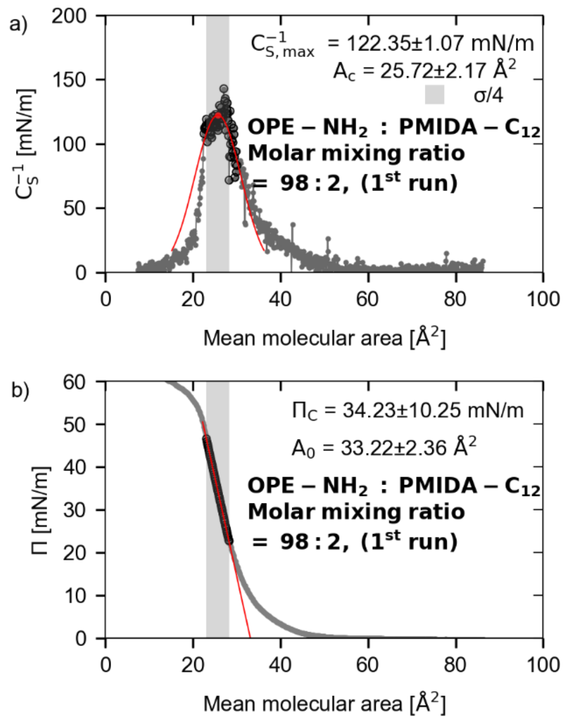

Figure S27: Analysis of first individual Π(mma) isotherm of OPE-NH$_2$: PMIDA-C$_{12}$ mixture with molar mixing ratio [%] 98:2.

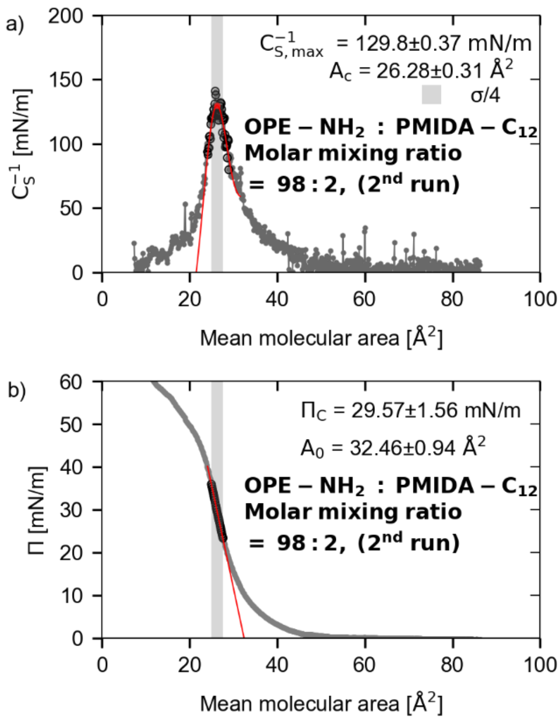

Figure S28: Analysis of second individual Π(mma) isotherm of OPE-NH$_2$: PMIDA-C$_{12}$ mixture with molar mixing ratio [%] 98:2.

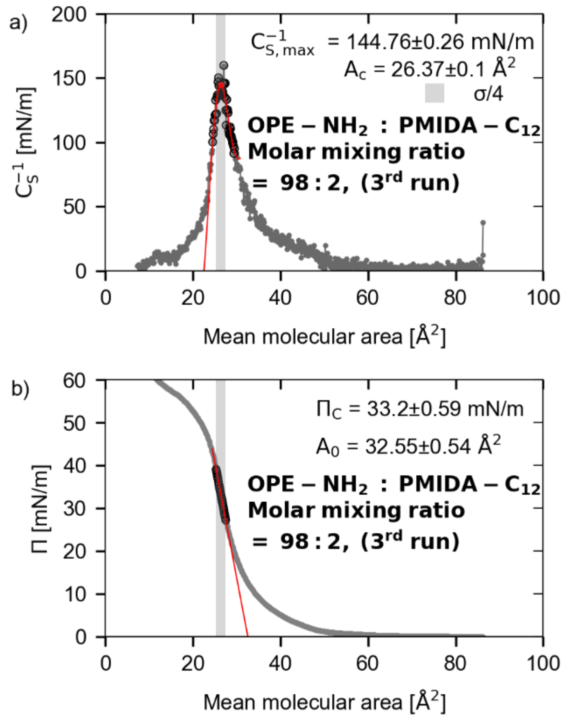

Figure S29: Analysis of third individual Π(mma) isotherm of OPE-NH$_2$: PMIDA-C$_{12}$ mixture with molar mixing ratio [%] 98:2.

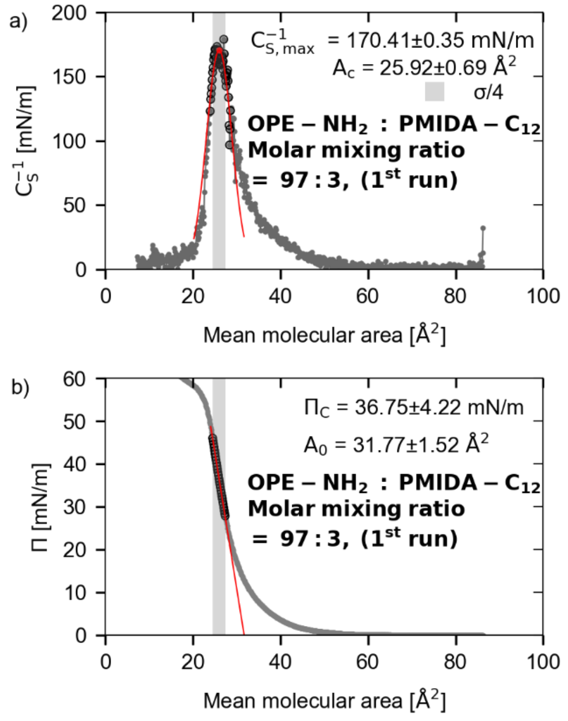

Figure S30: Analysis of first individual Π(mma) isotherm of OPE-NH$_2$: PMIDA-C$_{12}$ mixture with molar mixing ratio [%] 97:3.

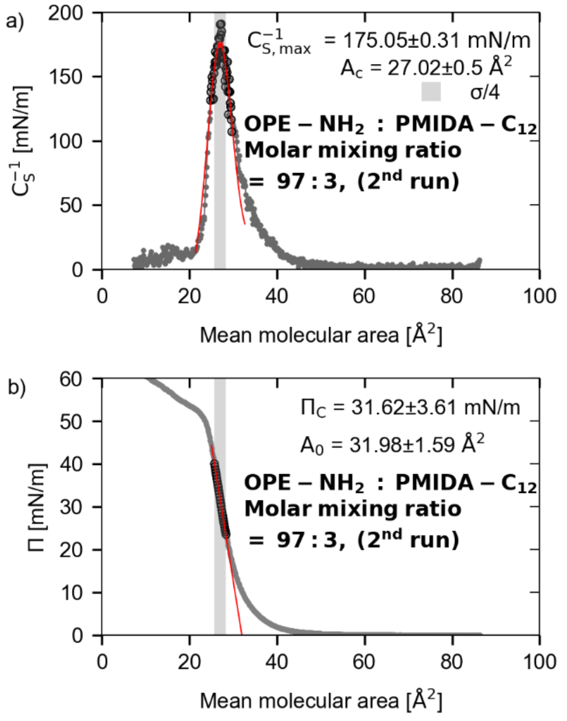

Figure S31: Analysis of second individual Π(mma) isotherm of OPE-NH$_2$: PMIDA-C$_{12}$ mixture with molar mixing ratio [%] 97:3.

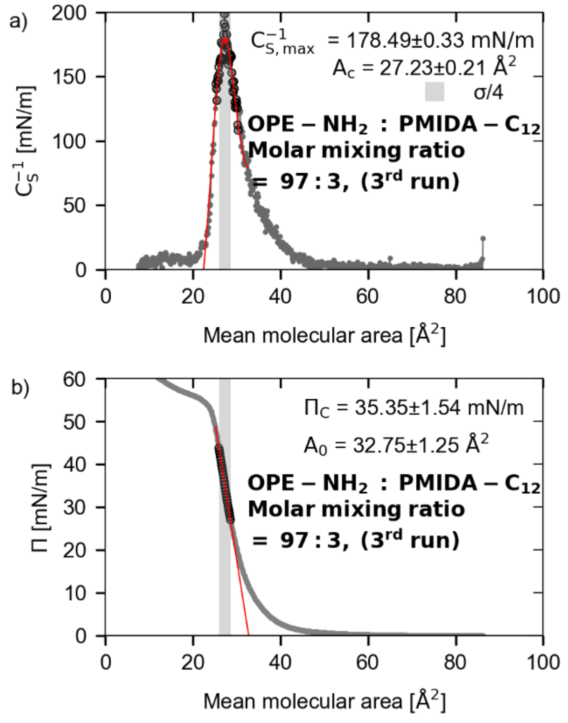

Figure S32: Analysis of third individual Π(mma) isotherm of OPE-NH$_2$: PMIDA-C$_{12}$ mixture with molar mixing ratio [%] 97:3.

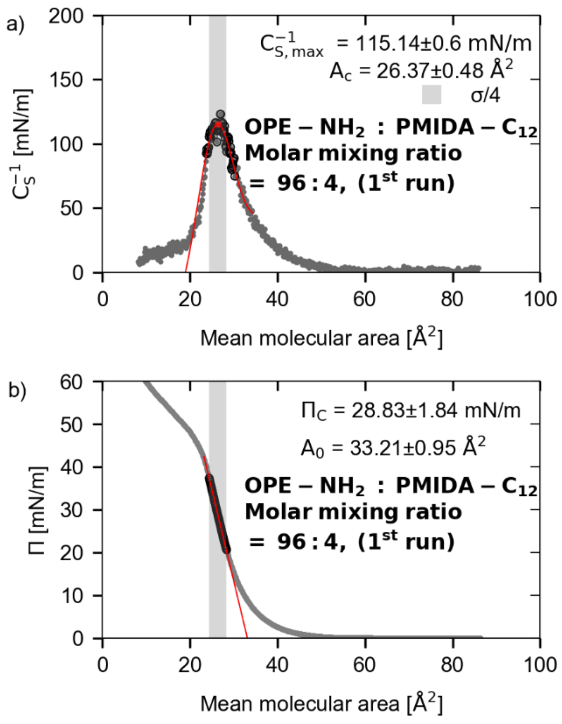

Figure S33: Analysis of first individual Π(mma) isotherm of OPE-NH$_2$: PMIDA-C$_{12}$ mixture with molar mixing ratio [%] 96:4.

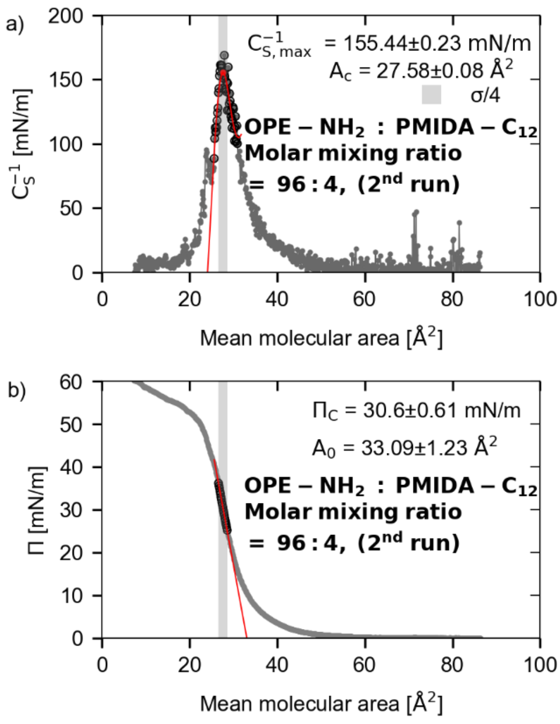

Figure S34: Analysis of second individual Π(mma) isotherm of OPE-NH$_2$: PMIDA-C$_{12}$ mixture with molar mixing ratio [%] 96:4.

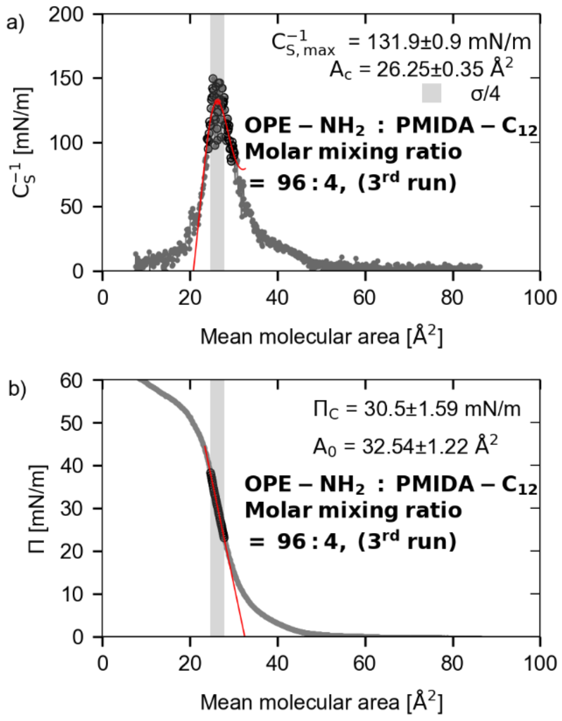

Figure S35: Analysis of third individual Π(mma) isotherm of OPE-NH$_2$: PMIDA-C$_{12}$ mixture with molar mixing ratio [%] 96:4.

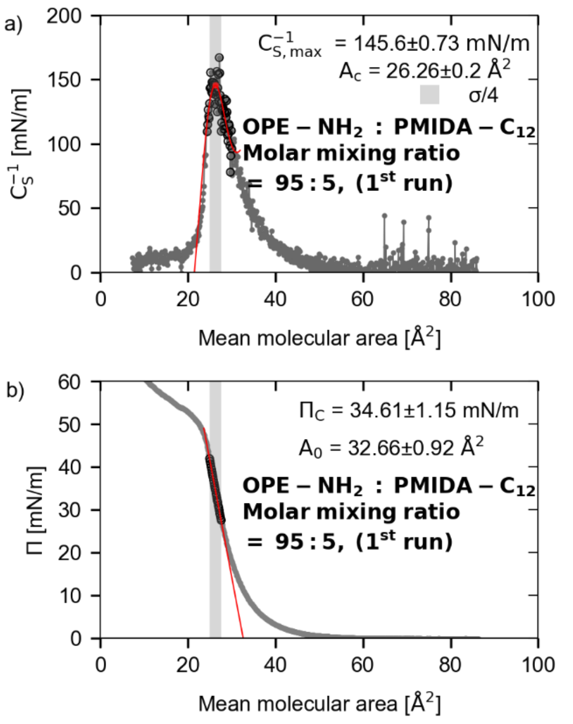

Figure S36: Analysis of first individual Π(mma) isotherm of OPE-NH$_2$: PMIDA-C$_{12}$ mixture with molar mixing ratio [%] 95:5.

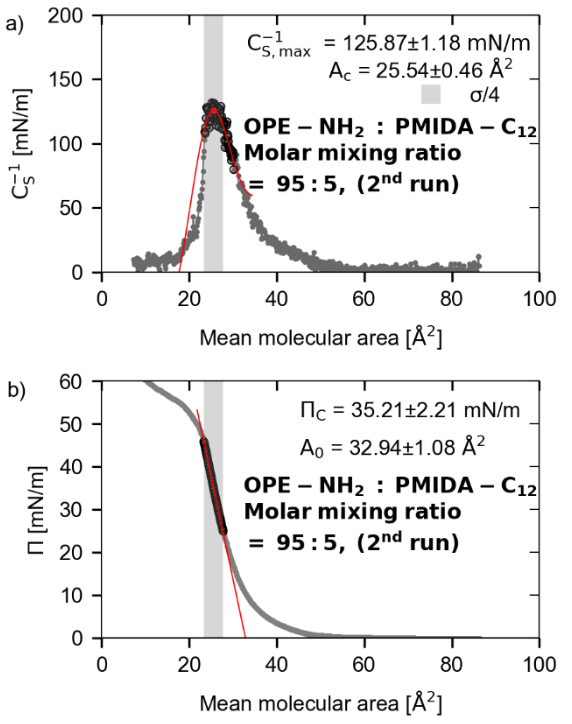

Figure S37: Analysis of second individual Π(mma) isotherm of OPE-NH$_2$: PMIDA-C$_{12}$ mixture with molar mixing ratio [%] 95:5.

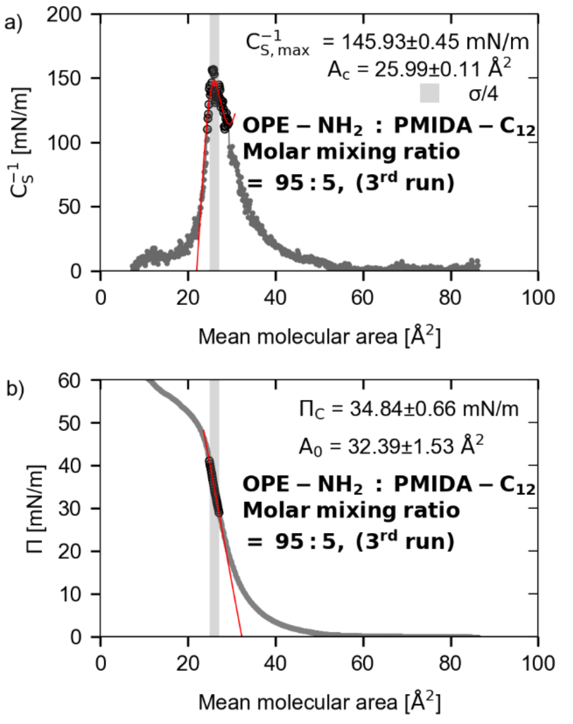

Figure S38: Analysis of third individual Π(mma) isotherm of OPE-NH$_2$: PMIDA-C$_{12}$ mixture with molar mixing ratio [%] 95:5.

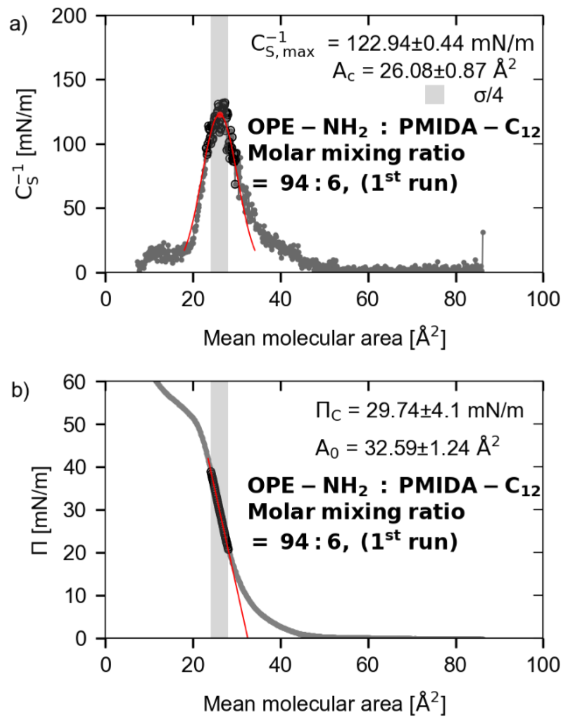

Figure S39: Analysis of first individual Π(mma) isotherm of OPE-NH$_2$: PMIDA-C$_{12}$ mixture with molar mixing ratio [%] 94:6.

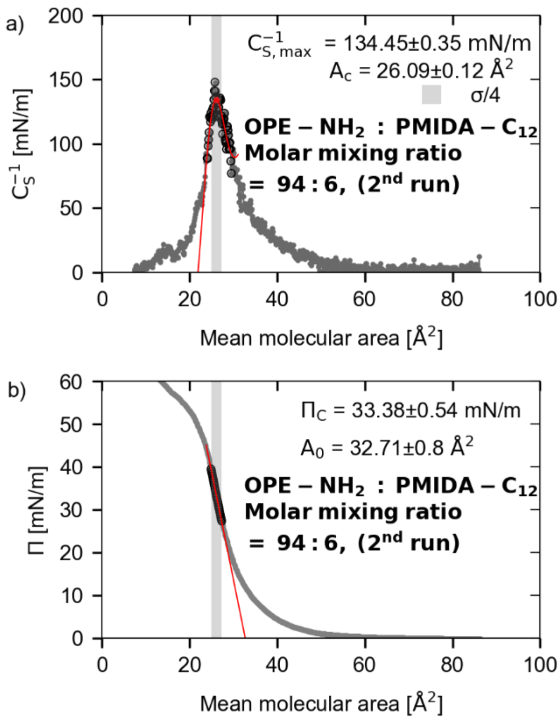

Figure S40: Analysis of second individual Π(mma) isotherm of OPE-NH$_2$: PMIDA-C$_{12}$ mixture with molar mixing ratio [%] 94:6.

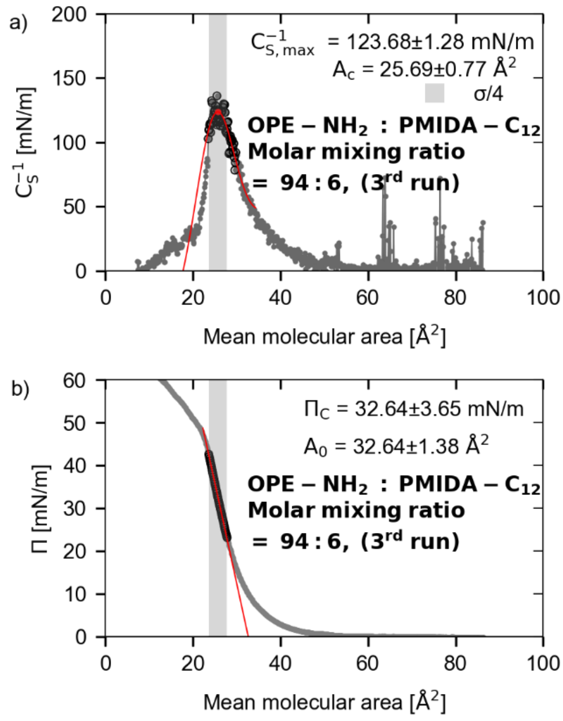

Figure S41: Analysis of third individual Π(mma) isotherm of OPE-NH$_2$: PMIDA-C$_{12}$ mixture with molar mixing ratio [%] 94:6.

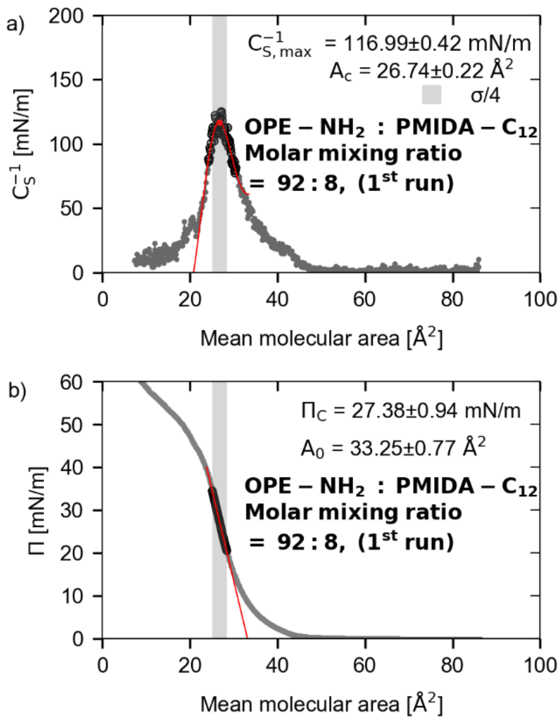

Figure S42: Analysis of first individual Π(mma) isotherm of OPE-NH$_2$: PMIDA-C$_{12}$ mixture with molar mixing ratio [%] 92:8.

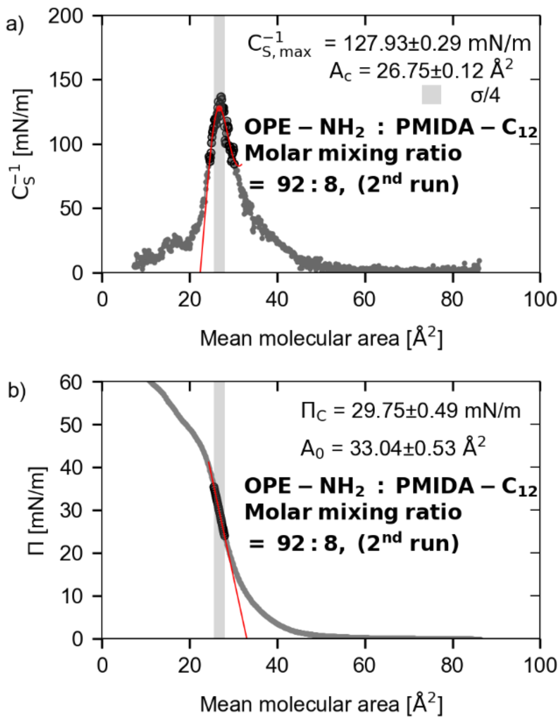

Figure S43: Analysis of second individual Π(mma) isotherm of OPE-NH$_2$: PMIDA-C$_{12}$ mixture with molar mixing ratio [%] 92:8.

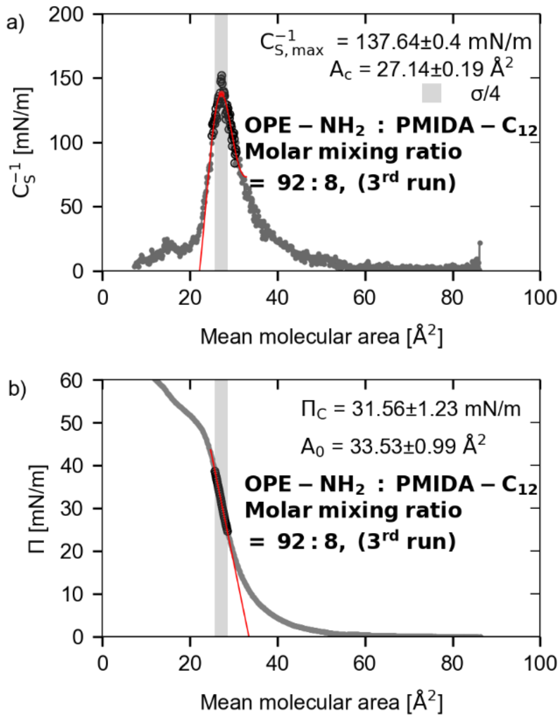

Figure S44: Analysis of third individual Π(mma) isotherm of OPE-NH$_2$: PMIDA-C$_{12}$ mixture with molar mixing ratio [%] 92:8.

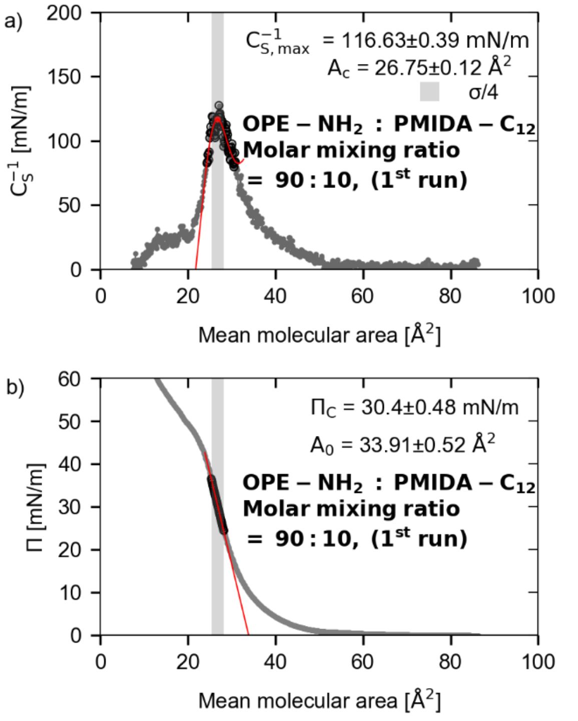

Figure S45: Analysis of first individual Π(mma) isotherm of OPE-NH$_2$: PMIDA-C$_{12}$ mixture with molar mixing ratio [%] 90:10.

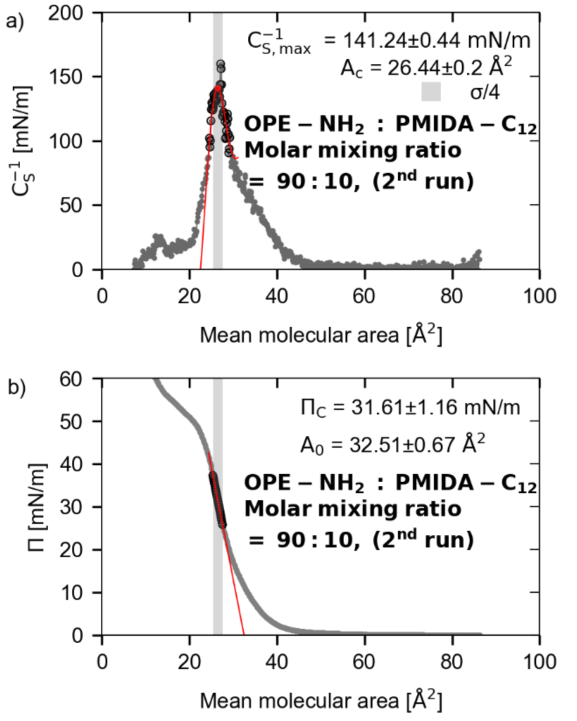

Figure S46: Analysis of second individual Π(mma) isotherm of OPE-NH$_2$: PMIDA-C$_{12}$ mixture with molar mixing ratio [%] 90:10.

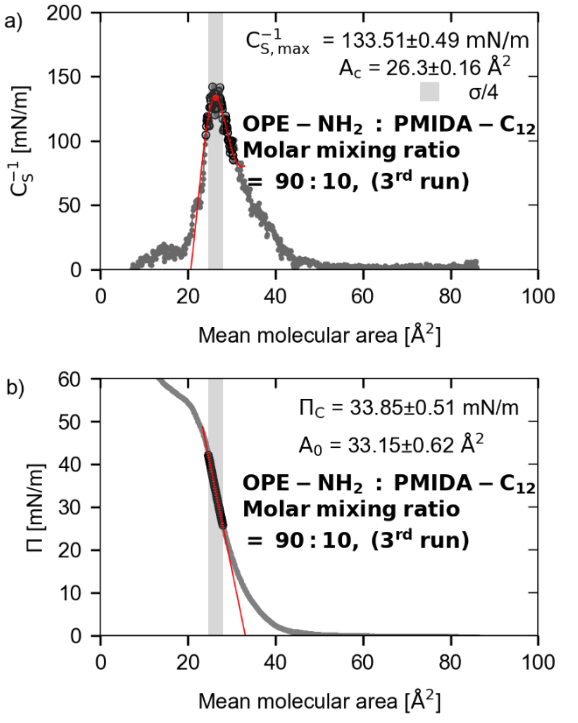

Figure S47: Analysis of third individual Π(mma) isotherm of OPE-NH$_2$: PMIDA-C$_{12}$ mixture with molar mixing ratio [%] 90:10.

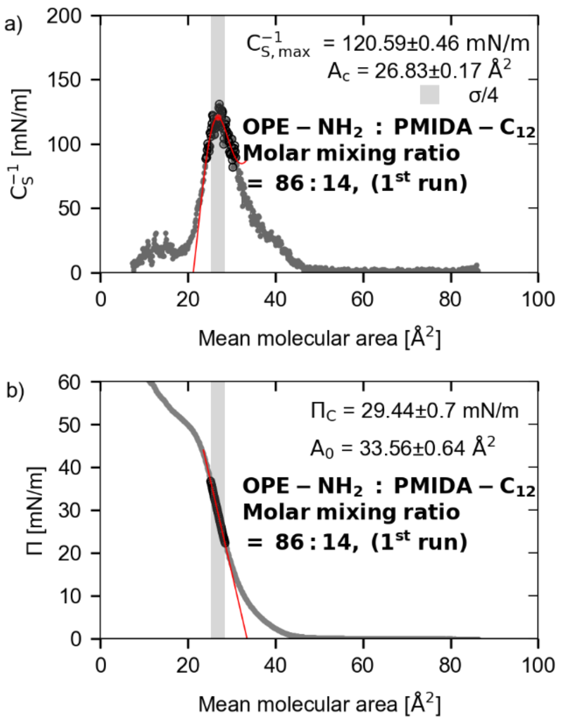

Figure S48: Analysis of first individual Π(mma) isotherm of OPE-NH$_2$: PMIDA-C$_{12}$ mixture with molar mixing ratio [%] 86:14.

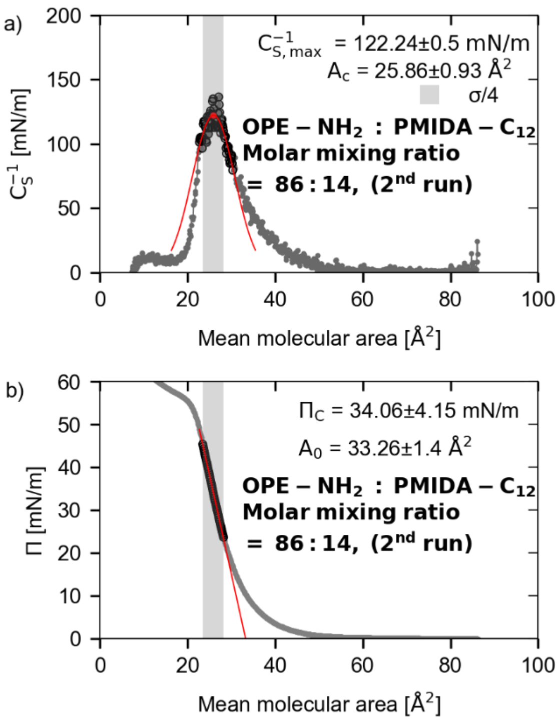

Figure S49: Analysis of second individual Π(mma) isotherm of OPE-NH$_2$: PMIDA-C$_{12}$ mixture with molar mixing ratio [%] 86:14.

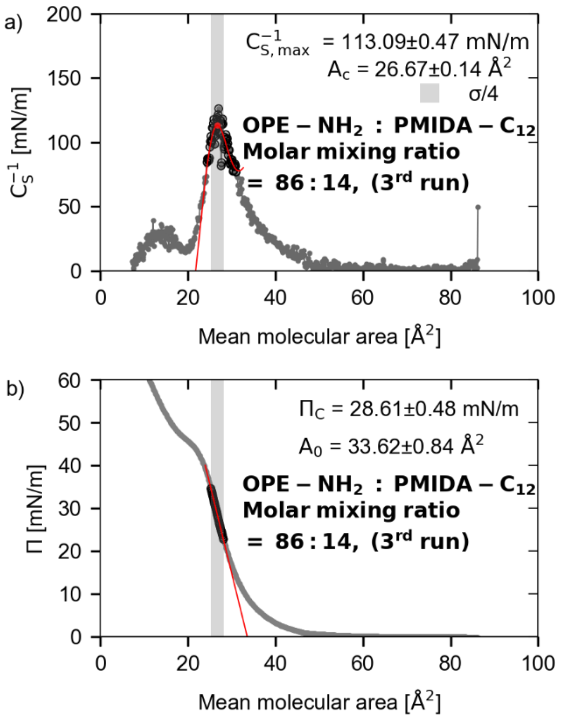

Figure S50: Analysis of third individual Π(mma) isotherm of OPE-NH$_2$: PMIDA-C$_{12}$ mixture with molar mixing ratio [%] 86:14.

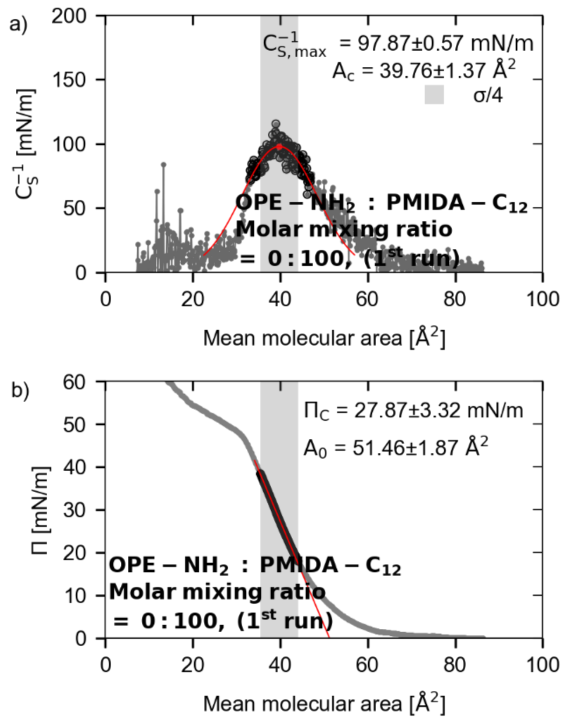

Figure S51: Analysis of first individual Π(mma) isotherm of pristine PMIDA-C_{12}.

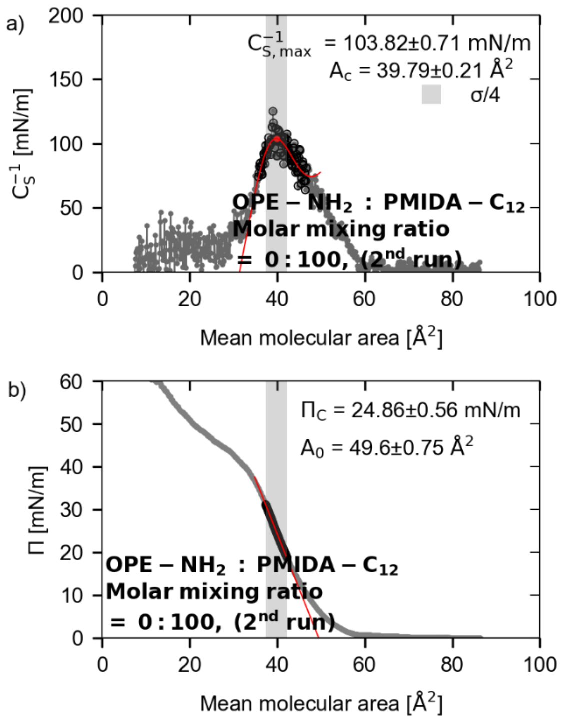

Figure S52: Analysis of second individual Π(mma) isotherm of pristine PMIDA-C_{12}.

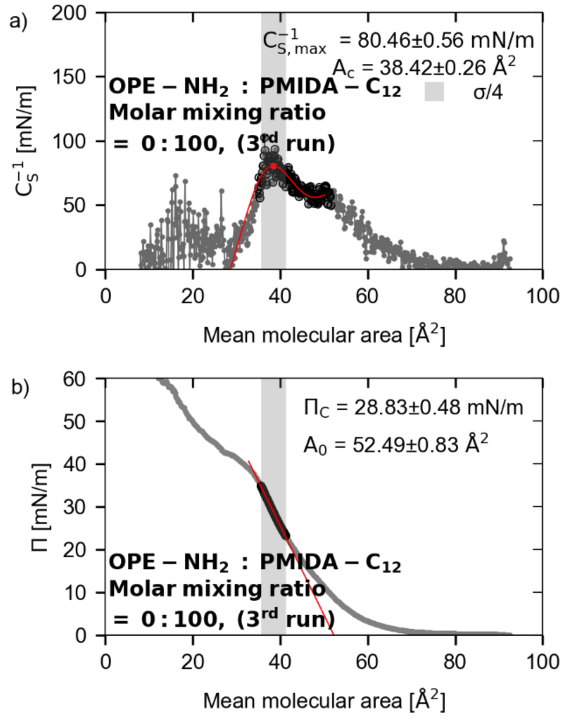

Figure S53: Analysis of third individual Π(mma) isotherm of pristine PMIDA-C_{12}.

4. Determination of space requirement for OPE molecules via aggregation

For the space requirement analysis for OPE molecules, we have generated quadrumers using the technique, which were introduced in our previous work for aggregation of pi-stacked molecules.[15] Starting from pre-oriented monomers, we enumerated rigid-body dimer poses on a Cartesian grid of translations and rotations, applied a vdW-based clash filter and deduplicated candidates by heavy-atom Kabsch RMSD (nRMSD). From the best dimer poses we greedily extended to quadrumers by resampling local translations/rotations around the accepted geometry, enforcing minimum inter-plane separation to favor π-stacking–like arrangements.

After their generation the conformers are grouped into geometrically similar structures using hierarchical clustering. This bottom-up approach starts by treating each conformational structure as an individual cluster. Subsequently, the nearest clusters are progressively merged into larger clusters until a single all-encompassing cluster remains. All dimer geometries were optimized with density functional theory calculations with CAM-B3LYP/lanl2dz,[16, 17] Grimme's D3 dispersion correction,[18, 19] and the Conductor-like Screening Model (COSMO) in the conductor limit.[20, 21] The resulting molecular structures are subsequently sorted based on their energies and structural similarity. Quantum chemical structure optimizations and single point calculations were performed using density functional theory (DFT) as implemented in the GPU-accelerated program TeraChem.[22-25]

The dimers that were found to be unique and energetically favorable according to Figure S54 were namely:

- Dimer 2 with translations over x, y, z = (−2.844, 0.704, −0.435) with ‖t‖ ≈ 2.96 Å as the shift between the two monomer reference planes/frames. The planes of the molecules are T-shaped.
- Dimer4 with translations (2.030, 0.482, −0.016) and with ‖t‖ ≈ 2.09 Å, giving a tighter, more vertical offset. The molecules in the dimer parallel shifted.

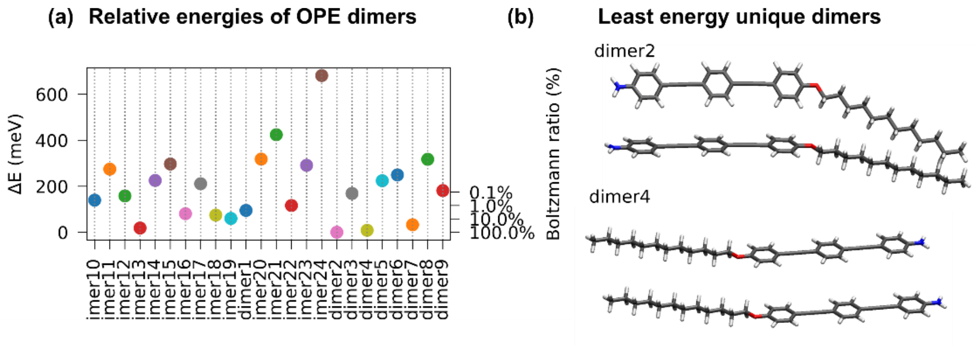

Figure S54: Relative energies (ΔE, meV) of candidate dimers referenced to the global minimum (left; marker size encodes the Boltzmann population at 298 K shown on the right axis). Representative low-energy geometries are shown for dimer 2 and dimer 4 (right). These two motifs dominate the room-temperature ensemble among the lowest-ΔE structures.

We selected the two lowest-energy motifs—dimer 2 and dimer 4—as building blocks and generated quadrumers from all ordered pairings of these dimers (2–2, 2–4, 4–2, 4–4). Each quadrumer was constructed with the same placement protocol used for dimers: grid-based relative translations/rotations of the incoming unit, rigid-body assembly with clash (vdW) rejection and minimum-distance checks.

We optimized all exported quadrumer geometries with GFN2-xTB tight-binding method (self-consistent extended tight binding Hamiltonian with isotropic second order electrostatic contributions and third order on-site contributions)[26] in the vacuum environment. For each input structure we have invoked xtb and the integral/SCF accuracy was set to 1.0, and the maximum number of geometry steps was limited to 20. After each run, the final optimized coordinates and energies reported by xtb were collected for analysis. All xTB calculations were executed via the xtb-python API.

Only aggregates within 250 meV of the global optimum (ΔE=0 eV) were considered favorable, as those exceeding this threshold are almost negligible (observed with approx. 0.005% probability) according to the Boltzmann distribution ($\frac{p_i}{p_0} = e^{-\frac{(E_i-E_0)}{kT}}$). Thus, aggregates beyond 250 meV can be ignored. Clustering algorithm was used for resampling of aggregates, and an RMSD matrix was presented for the found structures. Pairwise RMSD between each two aggregates is calculated using Kabsch algorithm.[27]

We clustered all optimized quadrumer geometries by heavy-atom shape similarity using a Kabsch-aligned, normalized RMSD (nRMSD) metric and a two-threshold scheme to separate coarse partitions from fine families. This produces a symmetric distance matrix used for agglomerative clustering (Ward linkage by default).

We apply:

- a BIG nRMSD threshold (0.2 Å) to partition the dendrogram into coarse clusters, and
- within each BIG cluster, a SMALL threshold (default 0.03 Å) to define finer families (color groups).

For each BIG cluster, we also output a local dendrogram (same coloring/limits) and an energy scatter in dendrogram order, colored by SMALL families with filled markers denoting the lowest-energy member within each SMALL family (energies are relative to the global minimum). The big cluster results for quadrumers, made from dimers 2, 4 and their combination are presented in Figure S55 to Figure S57. One can also see, that as the same translational and rotational grid was applied for different dimer combinations, the groupings for the quadrumers and local minima were reproduced throughout all three sets of quadrumers.

After the two-threshold nRMSD clustering, we performed a standardized follow-up to assemble a non-redundant set of energetically favorable dimers directly from the original folders used for quadrumer generation. Within every SMALL family, we selected the single lowest-energy member subject to a relative-energy cutoff of ΔE < 250 meV. The chosen quadrumer file was copied into a merged collection and re-labeled as dimerK_A_B.xyz, where <A>_<B> encodes the source pairing and K is a running index.

For the merged set, we rebuilt a global energy table. Global relative energies were recomputed against the overall minimum of the merged pool. We then recalculated shape similarity via heavy-atom Kabsch nRMSD (RMSD divided by the heavy-atom count), produced the pairwise distance matrix, and applied agglomerative clustering (default Ward linkage; alternatives available). Then the energetically favourable, distinct quadrumers are selected only for further analysis.

For each selected quadrumer geometry, we estimate the 2D “footprint” area spanned by the four molecules. Each molecule contributes a single setting point—its center of mass (COM)—and the area is the polygon enclosed by these four COMs after projection to their best-fit plane.

Best-fit plane (PCA). The four COMs are centered and a PCA is performed; the two principal axes span the projection plane. Practically, we compute the 3×3 covariance of the centered points and take its eigenvectors. The eigenvector with the smallest eigenvalue is the normal; the other two eigenvectors form an orthonormal basis for the plane. he best-fit plane is the set of points whose normal component (along that smallest-spread direction) equals zero at the centroid. It is the unique plane that minimizes the total squared perpendicular distances from all input points.

The 2D points are ordered counter-clockwise (CCW) by polar angle around their 2D centroid. The polygon area is then estimated with the shoelace formula.

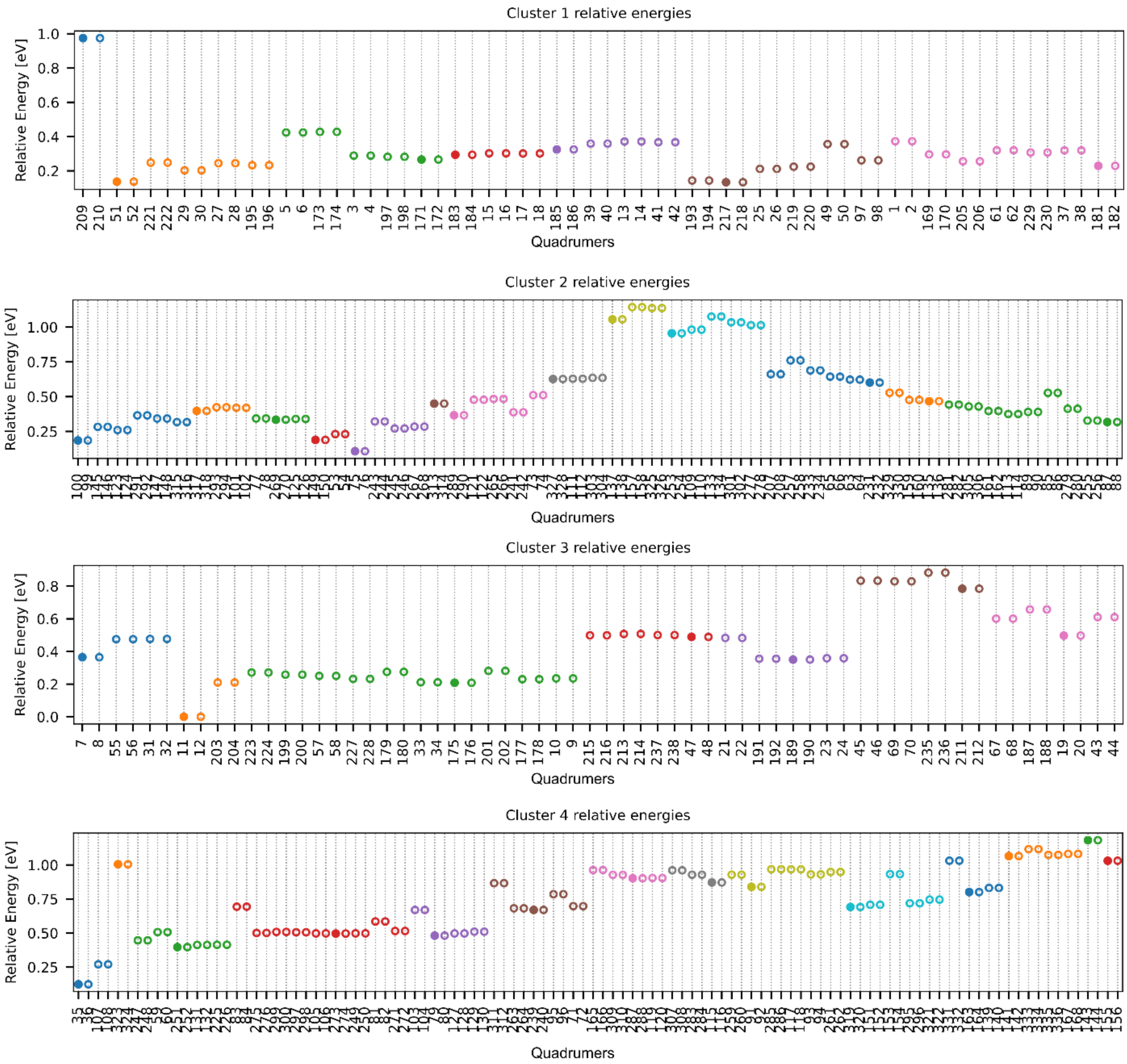

Figure S55: Relative energies (ΔE, meV) within each hierarchical BIG partition of the quadrumer set made up from two dimers 4 (BIG cut = 0.2 Å nRMSD; Ward linkage on heavy-atom Kabsch nRMSD). Panels show BIG clusters c1–c4 in their local dendrogram order (x-axis: numeric quadrumer labels; dotted lines are visual guides). Points are colored by SMALL families (nRMSD < 0.03 Å); filled markers indicate the lowest-energy member of each SMALL family. Energies are referenced to the global minimum across all dimers. These per-cluster landscapes were used to pick energy representatives for further quadrumer comparison.

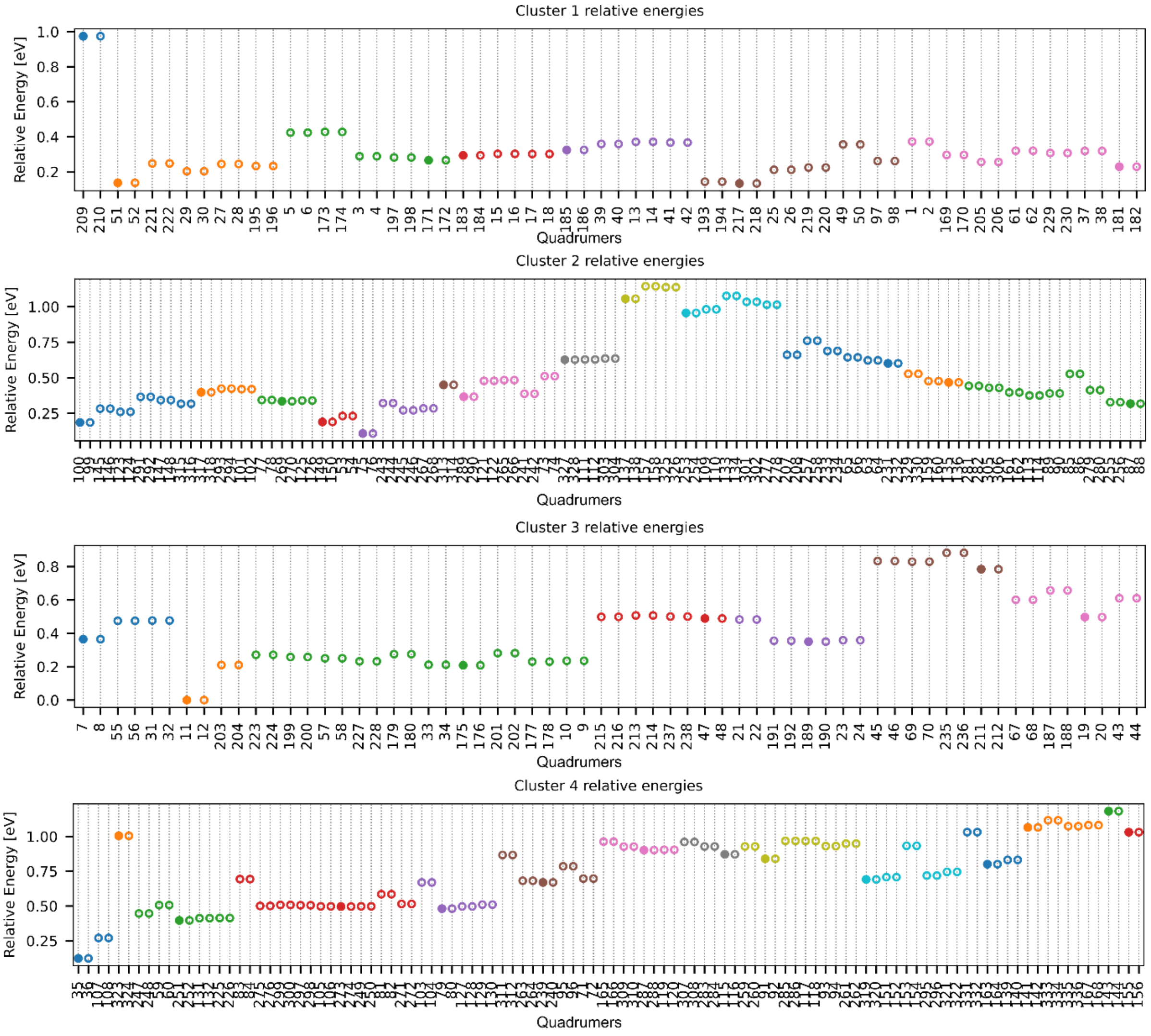

Figure S56: Relative energies (ΔE, meV) within each hierarchical BIG partition of the quadrumer set made up from dimer 2 and dimer 4. Panels show BIG clusters c1–c4 in their local dendrogram order. Points are colored by SMALL families (nRMSD < 0.03 Å); filled markers indicate the lowest-energy member of each SMALL family. Energies are referenced to the global minimum across all dimers.

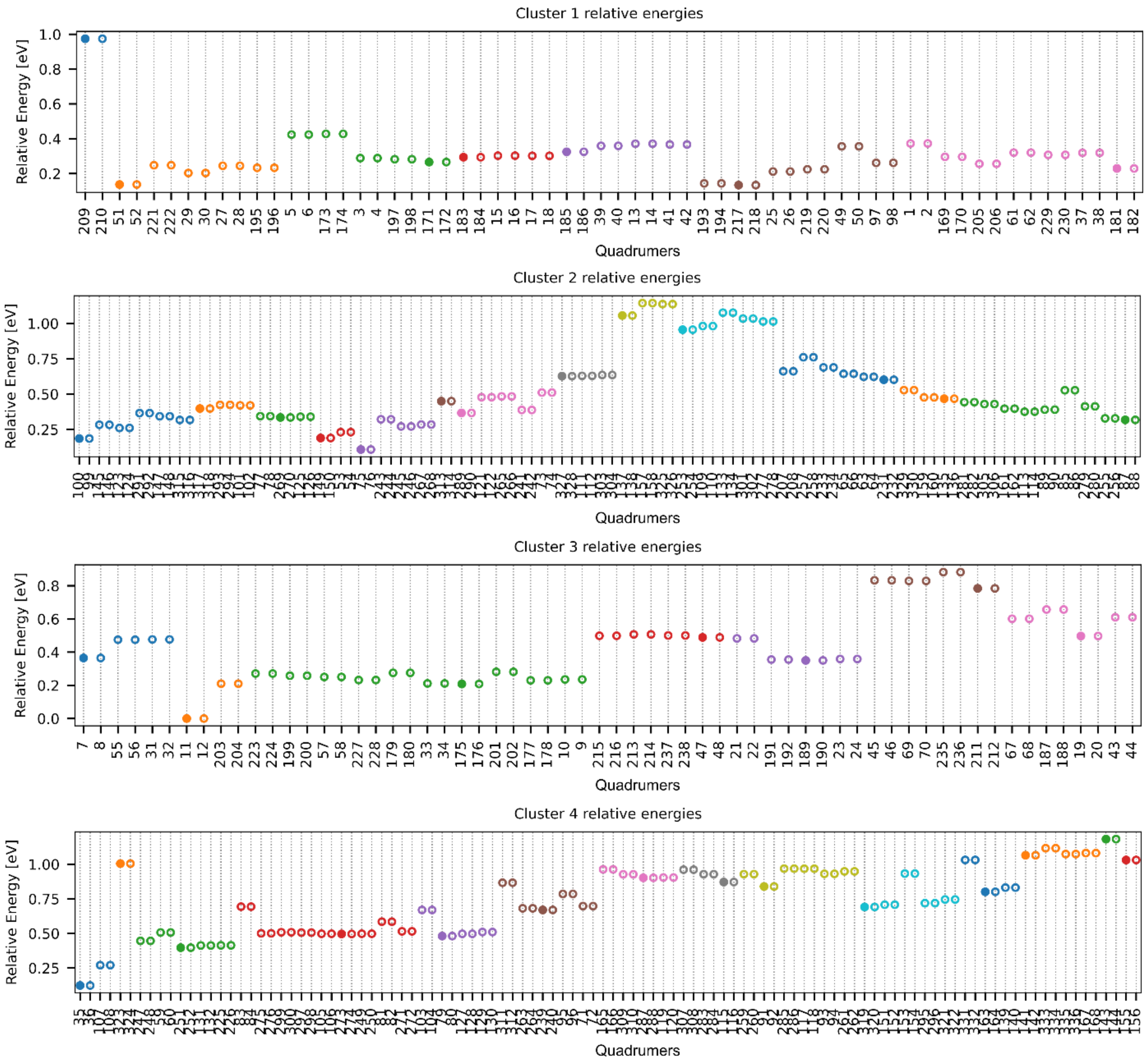

Figure S57: Relative energies (ΔE, meV) within each hierarchical BIG partition of the quadrumer set made up from two dimers 4. Panels show BIG clusters c1–c4 in their local dendrogram order. Points are colored by SMALL families (nRMSD < 0.03 Å); filled markers indicate the lowest-energy member of each SMALL family. Energies are referenced to the global minimum across all dimers.

5. NMR spectra

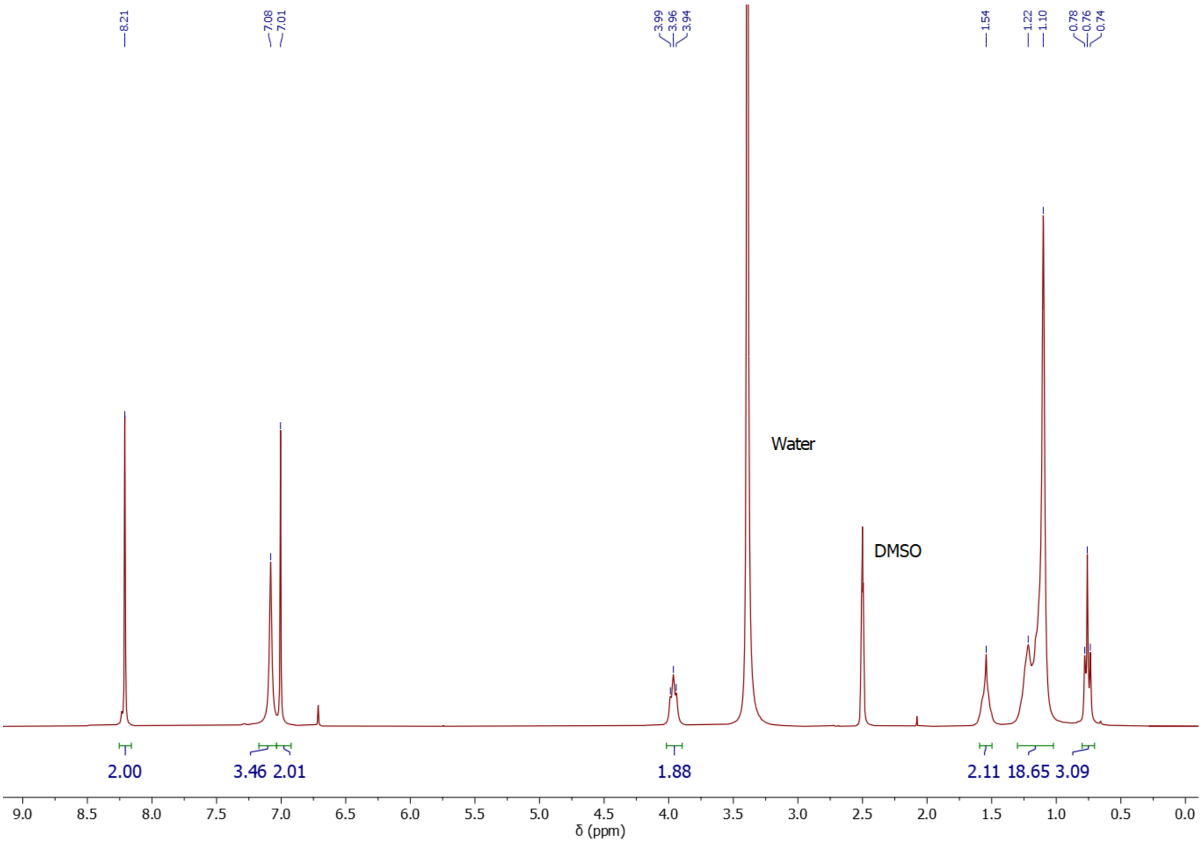

*Figure S57: ^{1}H-NMR spectra of **PMIDA-C$_{12}$** in DMSO-d$_6$ (300 MHz; 300 K).*

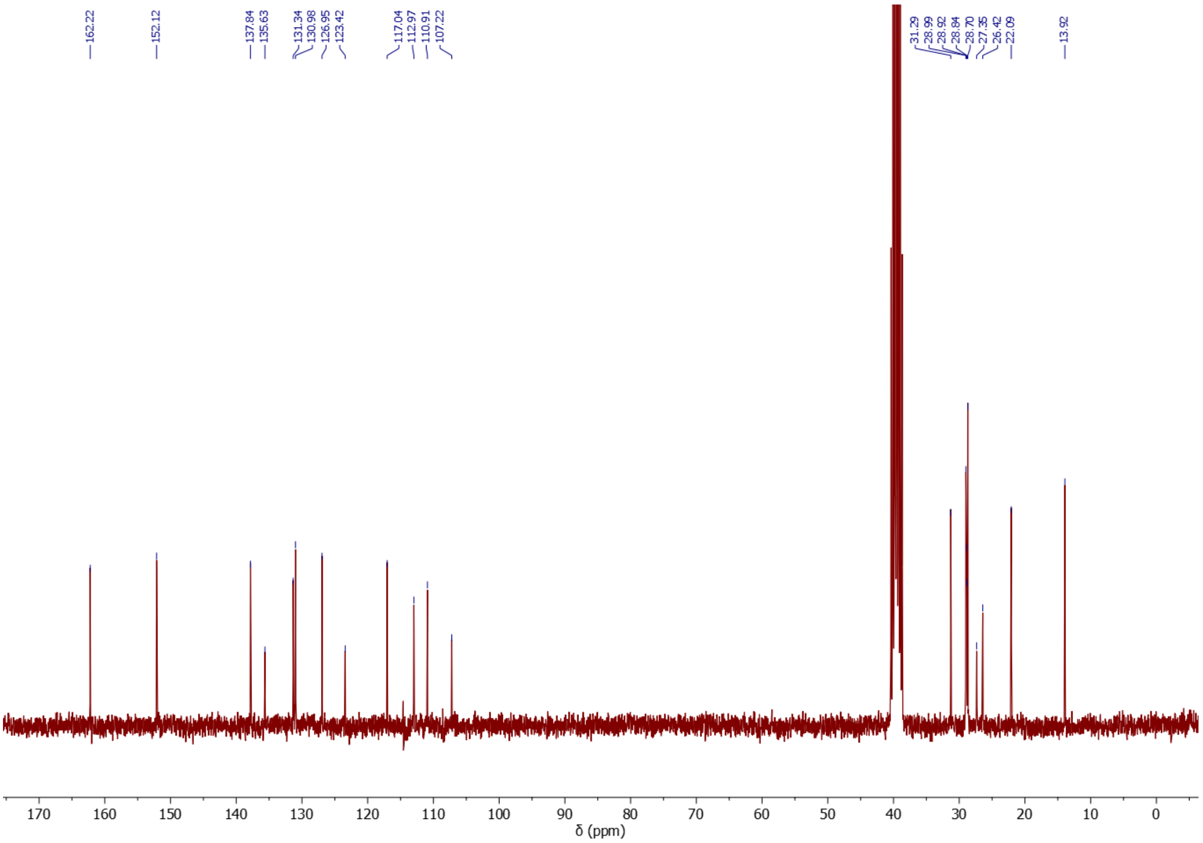

*Figure S58: ^{13}C-NMR spectra of **PMIDA-C$_{12}$** in DMSO-d$_6$ (75 MHz; 300 K).*

References

(1) Finkelmeyer, S. J.; Mankel, C.; Ansay, G.; Elmanova, A.; Zechel, S.; Martin D. Hager; Schubert, U. S.; Presselt, M. Filling the gaps: Introducing plasticizers into π-conjugated OPE-NH2 Langmuir layers for defect-free anisotropic interfaces and membranes towards unidirectional mass, charge, or energy transfer. *J. Colloid Interface Sci.* **2025**, *680*, 1090-1100. DOI: 10.1016/j.jcis.2024.11.020.
(2) Kluyver, T.; Ragan-Kelley, B.; Pérez, F.; Granger, B.; Bussonnier, M.; Frederic, J.; Kelley, K.; Hamrick, J.; Grout, J.; Corlay, S.; et al. Jupyter notebooks—a publishing format for reproducible computational workflows. In *IOS Press*, 2016; pp 87-90.
(3) Granger, B. E.; Pérez, F. Jupyter: Thinking and storytelling with code and data. *Comput. Sci. Eng.* **2021**, *23* (2), 7-14. DOI: 10.1109/MCSE.2021.3059263.
(4) Rossum, G. V.; Drake, F. L. *Python 3 Reference Manual*; CreateSpace, 2009.
(5) Nakahara, H.; Ohmine, A.; Kai, S.; Shibata, O. Monolayer compression induces fluidization in binary system of partially fluorinated alcohol (F4H11OH) with DPPC. *Journal of Oleo Science* **2013**, *62* (5), 271-281. DOI: 10.5650/jos.62.271.
(6) Hupfer, M. L.; Blaschke, D.; Schmidt, H.; Presselt, M. Embedding an amphiphilic 4-hydroxy thiazole dye in Langmuir matrices: Studying miscibilities with arylic and alkylic matrix amphiphiles via Langmuir isotherms and photo-induced force microscopy. *Langmuir* **2021**, *37* (45), 13255-13264. DOI: 10.1021/acs.langmuir.1c01772.
(7) Gavin, H. P. The Levenberg-Marquardt method for nonlinear least squares curve-fitting problems. 2022; Duke University: Vol. Department of Civil and Environmental Engineering.
(8) Huaren, Z.; Li, W. A modified Levenberg-Marquardt method with trust-region techniques. *Journal on Numerical Methods and Computer Applications* **2009**, *30*.
(9) Fan, B.; Ma, C.; Wu, A.; Wu, C. A Levenberg–Marquardt method for nonlinear complementarity problems based on nonmonotone trust region and line search techniques. *Mediterranean Journal of Mathematics* **2018**, *15* (3), 118. DOI: 10.1007/s00009-018-1168-y.
(10) Krejić, N.; Malaspina, G.; Swaenen, L. A split Levenberg-Marquardt method for large-scale sparse problems. *Computational Optimization and Applications* **2023**, *85* (1), 147-179. DOI: 10.1007/s10589-023-00460-9.
(11) Lourakis, M. A brief description of the Levenberg-Marquardt algorithm implemened by levmar. *Foundation for Research and Technology - Hellas (FORTH), Heraklion, GREECE* **2005**.
(12) Moré, J. J. The Levenberg-Marquardt algorithm: Implementation and theory. In *Numerical Analysis*, Berlin, Heidelberg, 1978; Watson, G. A., Ed.; Springer Berlin Heidelberg: pp 105-116. DOI: 10.1007/BFb0067700.
(13) Nocedal, J.; Wright, S. *Numerical optimization*; Springer New York, 2006. DOI: 10.1007/978-0-387-40065-5.
(14) Ochoa, B. L.; Belongie, S. J. Covariance propagation for guided matching. 2006.
(15) Elmanova, A.; Jahn, B. O.; Presselt, M. Catching the π-stacks: Prediction of aggregate structures of porphyrin. *The Journal of Physical Chemistry A* **2024**, *128* (46), 9917-9926. DOI: 10.1021/acs.jpca.4c05969.
(16) Krishnan, R.; Binkley, J. S.; Seeger, R.; Pople, J. A. Self-consistent molecular orbital methods. XX. A basis set for correlated wave functions. *The Journal of Chemical Physics* **1980**, *72* (1), 650-654. DOI: 10.1063/1.438955.
(17) Katagi, T. AM1 study of acid-catalyzed hydrolysis of maleamic (4-amino-4-oxo-2-butenoic) acids. *J. Comput. Chem.* **1990**, *11* (9), 1094-1100. DOI: 10.1002/jcc.540110913.
(18) Grimme, S.; Hujo, W.; Kirchner, B. Performance of dispersion-corrected density functional theory for the interactions in ionic liquids. *Physical Chemistry Chemical Physics* **2012**, *14* (14), 4875-4883, 10.1039/C2CP24096C. DOI: 10.1039/C2CP24096C.
(19) Grimme, S.; Hansen, A.; Brandenburg, J. G.; Bannwarth, C. Dispersion-corrected mean-field electronic structure methods. *Chemical Reviews* **2016**, *116* (9), 5105-5154. DOI: 10.1021/acs.chemrev.5b00533.
(20) Klamt, A.; Schüürmann, G. COSMO: a new approach to dielectric screening in solvents with explicit expressions for the screening energy and its gradient. *Journal of the Chemical Society, Perkin Transactions 2* **1993**, (5), 799-805, 10.1039/P29930000799. DOI: 10.1039/P29930000799.
(21) Klamt, A. Conductor-like screening model for real solvents: A new approach to the quantitative calculation of solvation phenomena. *J Phys Chem-Us* **2002**, *99* (7), 2224-2235. DOI: 10.1021/j100007a062.
(22) Isborn, C. M.; Luehr, N.; Ufimtsev, I. S.; Martínez, T. J. Excited-state electronic structure with configuration interaction singles and tamm–dancoff time-dependent density functional theory on graphical processing units. *J. Chem. Theory Comput.* **2011**, *7* (6), 1814-1823. DOI: 10.1021/ct200030k.
(23) Titov, A. V.; Ufimtsev, I. S.; Luehr, N.; Martinez, T. J. Generating efficient quantum chemistry codes for novel architectures. *J. Chem. Theory Comput.* **2013**, *9* (1), 213-221. DOI: 10.1021/ct300321a.
(24) Song, C.; Wang, L.-P.; Martínez, T. J. Automated code engine for graphical processing units: Application to the effective core potential integrals and gradients. *J. Chem. Theory Comput.* **2016**, *12* (1), 92-106. DOI: 10.1021/acs.jctc.5b00790.
(25) Ufimtsev, I. S.; Martinez, T. J. Quantum chemistry on graphical processing units. 3. Analytical energy gradients, geometry optimization, and first principles molecular dynamics. *J. Chem. Theory Comput.* **2009**, *5* (10), 2619-2628. DOI: 10.1021/ct9003004.
(26) Grimme, S.; Bannwarth, C.; Shushkov, P. A robust and accurate tight-binding quantum chemical method for structures, vibrational frequencies, and noncovalent interactions of large molecular systems parametrized for all spd-block elements (Z = 1–86). *Journal of Chemical Theory and Computation* **2017**, *13* (5), 1989-2009. DOI: 10.1021/acs.jctc.7b00118.
(27) Kabsch, W. A solution for the best rotation to relate two sets of vectors. *Acta Crystallographica Section A* **1976**, *32* (5), 922-923. DOI: 10.1107/S0567739476001873.
